# Interfering ribonucleic acids that suppress expression of multiple unrelated genes

**DOI:** 10.1186/1472-6750-9-57

**Published:** 2009-06-16

**Authors:** Toby Passioura, Mary M Gozar, Amber Goodchild, Andrew King, Greg M Arndt, Michael Poidinger, Donald J Birkett, Laurent P Rivory

**Affiliations:** 1 Johnson & Johnson Research Pty Ltd, Sydney, Australia

## Abstract

**Background:**

Short interfering RNAs (siRNAs) have become the research tool of choice for gene suppression, with human clinical trials ongoing. The emphasis so far in siRNA therapeutics has been the design of one siRNA with complete complementarity to the intended target. However, there is a need for multi-targeting interfering RNA in diseases in which multiple gene products are of importance. We have investigated the possibility of using a single short synthetic duplex RNA to suppress the expression of *VEGF-A *and *ICAM-1*; genes implicated in the progression of ocular neovascular diseases such as diabetic retinopathy.

**Results:**

Duplex RNA were designed to have incomplete complementarity with the 3'UTR sequences of both target genes. One such duplex, CODEMIR-1, was found to suppress VEGF and ICAM-1 by 90 and 60%, respectively in ARPE-19 cells at a transfected concentration of 40 ng/mL. Use of a cyan fusion reporter with target sites constructed in its 3'UTR demonstrated that the repression of VEGF and ICAM-1 by CODEMIR-1 was indeed due to interaction with the target sequence. An exhaustive analysis of sequence variants of CODEMIR-1 demonstrated a clear positive correlation between activity against VEGF (but not ICAM-1) and the length of the contiguous complementary region (from the 5' end of the guide strand). Various strategies, including the use of inosine bases at the sites of divergence of the target sequences were investigated.

**Conclusion:**

Our work demonstrates the possibility of designing multitargeting dsRNA to suppress more than one disease-altering gene. This warrants further investigation as a possible therapeutic approach.

## Background

The different triggers eliciting RNAi all ultimately lead to the formation of short (~21 nucleotide) RNA duplexes termed short interfering RNAs (siRNAs). Complete complementarity between the guide strand and the target mRNA leads to catalytic cleavage of the mRNA and suppresses gene expression [1]. Endogenous microRNAs (miRNAs) are also small duplex RNAs with diverse and critical roles in gene regulation [2]. miRNAs share many of the features of siRNAs including the loading of the guide strand into a RNA induced silencing complex (RISC) [3]. In contrast to siRNAs, mammalian miRNAs do not generally exhibit high complementarity to their cognate target sites. Binding of miRNAs to their target sites may induce target degradation or may prevent translation and reduce gene expression at the protein level [4]. In mammals, miRNAs are thought to bind to partially complementary sites predominantly located in the 3' untranslated regions (UTRs) of target mRNAs [2,4,5], thereby enabling the coordinate regulation of genes containing such sites.

Whilst the factors affecting siRNA activity have been extensively studied [3,6-8], the parameters affecting miRNA-mediated translational suppression have not yet been definitively elucidated. Binding of the 5' end of the guide strand to the target mRNA appears to be critical, with an almost absolute requirement for complementarity at the so-called "seed site" from positions 2–7 (measuring from the 5' end of the guide strand) [9-11]. miRNA target sites appear to be almost exclusively located in the 3'UTRs of target genes [11,12], and miRNA target sites may be functionally restricted to 3' UTRs, since binding of miRNAs to other sites in the transcript does not induce translational suppression.

The present study demonstrates proof of concept for the design of artificial short RNAs with at least partial complementarity to multiple unrelated transcripts, and which suppress the expression of the corresponding unrelated genes. The interfering RNAs described herein were designed to target expression of *VEGF-A *and *ICAM-1*, two genes involved in ocular neovascular disease [13]. We found that for suppression of *VEGF-A*, the length of complementarity to the seed region, as well as the total complementarity of the guide strand to the target were important determinants of activity. This relationship was not observed for *ICAM-1*, however this discrepancy appeared to result from specific sequence motifs in those guide strands with high *ICAM-1 *complementarity. Thus, the length of seed complementarity, and overall complementarity between the guide strand and the target should be considered in the design of multi-target interfering RNAs.

## Results

Transcript sequences corresponding to the 3' UTRs of *VEGF-A *and *ICAM-1 *(ensembl IDs ENST00000356655 and ENST00000264832, respectively) were used to search for a suitable seed of at least 6 contiguous bases present in both genes. A pool of all possible seeds of 6 bases or greater was generated using the specified length as a window and advancing the window in a stepwise fashion 1 base at a time. Low complexity seeds were eliminated and the pool was further restricted to those for which at least 3 contiguous bases were predicted to bind to an unpaired region in at least 50% of optimal and suboptimal (within -1 kcal/mol of optimal) folded structures (as determined using the Vienna RNA package [14]).

Two different seeds were selected for experimental testing: one of 12 bases in length and one of 7 bases that was within a genetic context that favoured the design of "consensus target sequences" (comprising the seed sequence and a consensus of the sequence adjacent to the seed sequence from the desired targets, in this instance, *VEGF-A *and *ICAM-1*). Multiple consensus target sequences were generated for both seeds by first aligning the target sequences (*VEGF-A *and *ICAM-1*) relative to their shared seed sequences. Then, extensions 5' to the seed sequences were proposed to a length of 21 nucleotides. The exact complements of these 21 nucleotide consensus target sequences were assessed *in silico *for hybridisation to the target sequences, and one sequence for each seed was selected for experimental testing. "Passenger" strands were designed to be complementary to the selected "guide" strands over a duplex length of 19 nt with 3' overhangs of 2 nucleotides (UU added in the case of the passenger strand).

Annealed RNA duplexes or single-stranded RNA oligonucleotides were purchased from Sigma-Proligo. Where required, 100 μM oligonucleotides were annealed in 50 μM Tris, 100 mM NaCl by heating to 90°C and cooling to 4°C over 3–4 hours. Control siRNA sequences are shown in Additional file 1.

**Figure 1:**
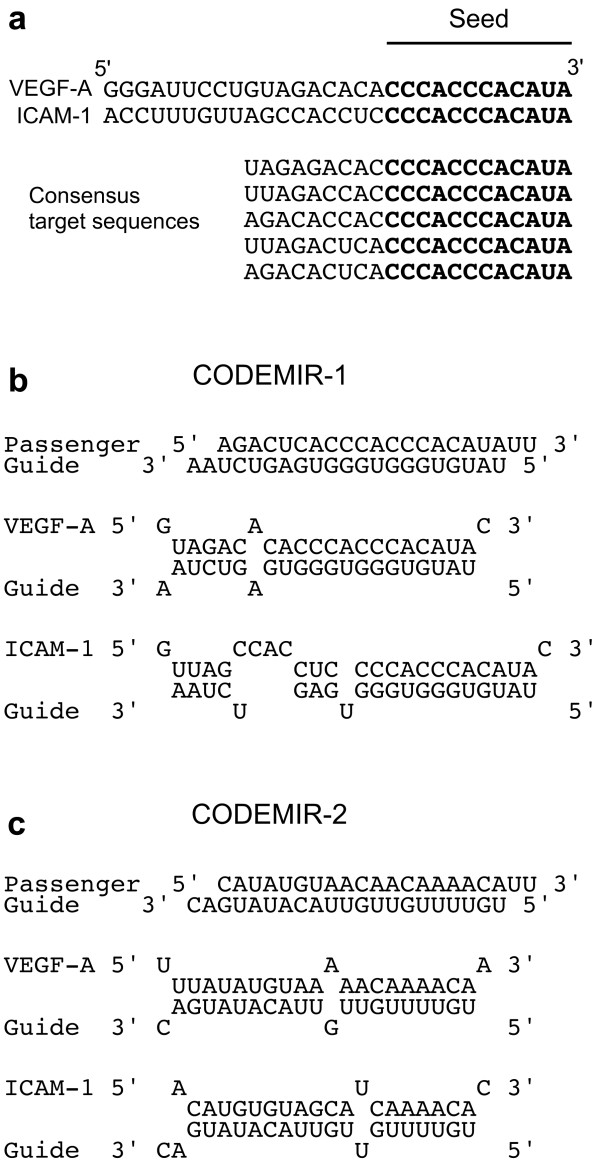
**Design of CODEMIRs targeting *VEGF-A *and *ICAM-1***. (a) Example of seed site alignment and consensus target sequence design for *VEGF-A *and *ICAM-1 *using a 12 nucleotide seed. (b) and (c) Schematic illustration of CODEMIR-1 and -2 (respectively) and guide strand binding to the *VEGF-A *and *ICAM-1 *mRNAs. Top strand represents the target mRNA (5' to 3'), bottom strand indicates the guide strand (3' to 5').

Because of the complexity of human disease, we sought to determine if it was possible to develop novel interfering RNA that can multi-target one or more pre-selected therapeutic targets. We achieved this by identifying short regions of homology in pre-selected target RNA with bioinformatic techniques. These "seeds" were then used to design short duplex RNAs having one strand that binds with at least partial complementarity to each target RNA sequence. Examples of such COmputationally DEsigned Multi-targeting Interfering RNAs (CODEMIRs) were sought to simultaneously target two genes (*VEGF-A *and *ICAM-1*) that are associated with ocular neovascular disease [13]. Two candidate seeds (7 and 12 contiguous bases) that are present in the 3' UTRs of both target mRNA were identified and used to design "consensus target sequences" (see Methods and Fig. 1a). The sequences complementary to these consensus target sequences (corresponding to the theoretical guide strands of CODEMIRs) were evaluated for binding to the target mRNAs using RNAhybrid software [15]. Two of these guide strands, one targeting each seed, were chosen for experimental testing; each having strong predicted binding to both target genes (Figs. 1b, c).

**Figure 2:**
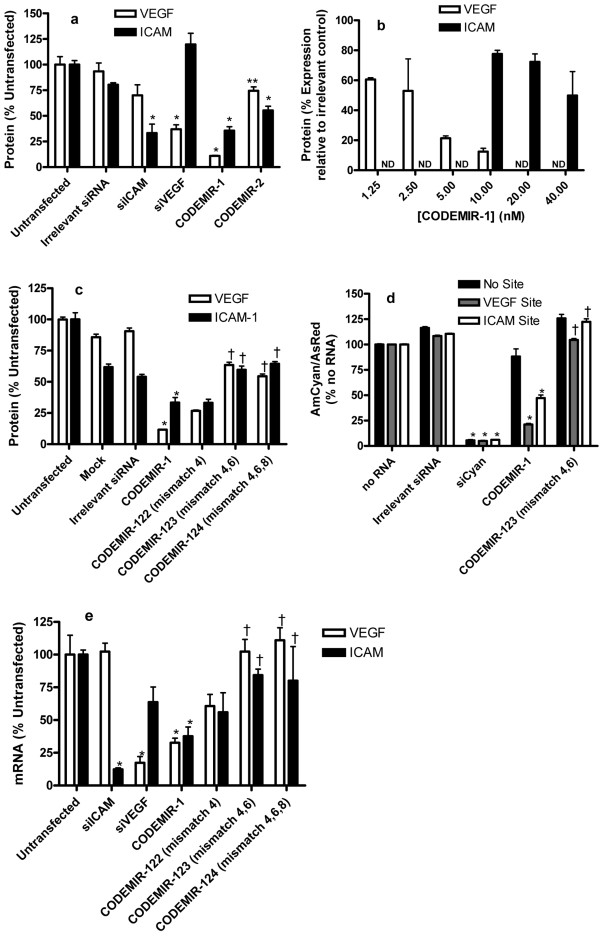
**Suppression of *VEGF-A *and *ICAM-1 *expression by CODEMIR-1 and -2**. Unless otherwise indicated, VEGF-A (ELISA) or ICAM-1 (FACS) were assayed in ARPE-19 cells 48 hours post-transfection and 24 hours post-stimulation with 130 μM Deferoxamine or 1 ng/mL IL-1β respectively. All data points indicate the mean of triplicate samples. Error bars show standard deviation. Statistical significance was determined by two-way ANOVA using a Bonferroni post-test (* p < 0.001 as compared to either untransfected and/or irrelevant siRNA; ** p < 0.001 as compared to untransfected and p < 0.01 as compared to irrelevant siRNA; † p < 0.001 compared to CODEMIR-1). (a) Gene suppression by CODEMIR-1 and -2. (b) Dose responsiveness of gene suppression by CODEMIR-1. Cells were transfected at the indicated concentrations. (ND = not determined). (c) Effect of mismatches on gene suppression by CODEMIR-1. (d) Suppression of *VEGF-A *and *ICAM-1 *reporter constructs by CODEMIR-1ARPE-19 cells were co-transfected with the AmCyan reporter and AsRed control plasmids and 40 nM indicated RNA duplexes. Fluorescence was assessed by FACS 48 hours post-transfection. (e) Suppression of *VEGF-A *and *ICAM-1 *mRNA expression by selected CODEMIRs. For *VEGF-A*, stimulation was performed 24 hours post-transfection using 65 μM Deferoxamine and the QuantiGene^® ^assay was performed on cell lysates prepared 24 hours post-stimulation. For *ICAM-1 *the QuantiGene^® ^assay was performed on cell lysates prepared 24 hours post-transfection.

Transfection of these CODEMIRs into the retinal epithelial cell line ARPE-19 induced significant suppression of both *VEGF-A *and *ICAM-1 *(Fig. 2a), demonstrating the feasibility of designing synthetic RNAs that suppress the expression of more than one gene at the protein level. The effects were qualitatively similar whether stimulation with deferoxamine or IL-1β was used or cells were cultured without stimulation. However, stimulation afforded a much greater dynamic range for the measurement of both VEGF-A and ICAM-1 enabling improved discrimination between CODEMIR designs. Thus, stimulation conditions were used in all experiments reported herein. CODEMIR-1 (which demonstrated greater efficacy than CODEMIR-2) was further characterised. CODEMIR-1 demonstrated dose-dependent suppression of VEGF-A and ICAM-1 expression in ARPE-19 cells (Fig. 2b). In contrast, the irrelevant cytokine IL-8, as measured by ELISA of culture supernatant (R&D Systems) was not affected by CODEMIR-1 under similar conditions, demonstrating the specificity of the effect. In order to confirm that seed binding was required for the activity of CODEMIR-1 against VEGF-A and ICAM-1, nucleotide substitutions introduced at positions 4, 4 and 6, or 4, 6 and 8 of the guide strand of CODEMIR-1 were tested in ARPE cells (Additional file 1). With the exception of CODEMIR-122 (position 4 mismatch), these had significantly impaired suppression of both *VEGF-A *and *ICAM-1 *relative to CODEMIR-1 at the protein level (Fig. 2c). Moreover, expression of a cyan fluorescent reporter gene containing the CODEMIR-1 target site in the 3'UTR was suppressed by CODEMIR-1 in a manner that correlated well with the observed suppression of endogenous *VEGF-A *and *ICAM-1 *(Fig. 2d), demonstrating that the observed suppression of *VEGF-A *and *ICAM-1 *was mediated by the binding of the guide strand of CODEMIR-1 to its predicted target sites. The above results all relate to expression at the protein level, however, at the mRNA level, CODEMIR-1 also caused significant suppression of both VEGF-A and ICAM-1, and this suppression was reduced by mismatches in the seed region (Fig. 2e).

**Figure 3:**
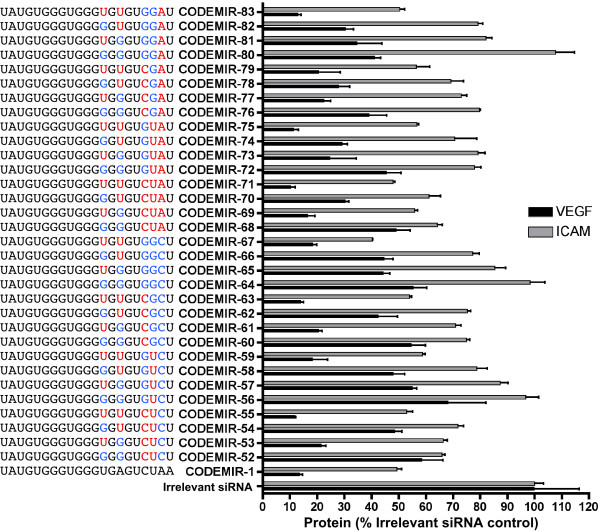
***VEGF-A *and *ICAM-1 *suppressive activity of 32 variants of CODEMIR-1**. ARPE-19 cells were transfected with 40 nM of the indicated RNA duplex, and VEGF-A (ELISA) and ICAM-1 (FACS) were assayed 48 hours post-transfection and 24 hours after stimulation with 130 μM Deferoxamine or 1 ng/mL IL-1β respectively. The guide strand of each CODEMIR is shown in the 5' to 3' direction, with blue bases indicating mismatches to the *VEGF-A *target sequence and red bases indicating mismatches to the *ICAM-1 *target sequence. Data points represent the mean of triplicate samples. Error bars indicate standard deviation.

**Figure 4:**
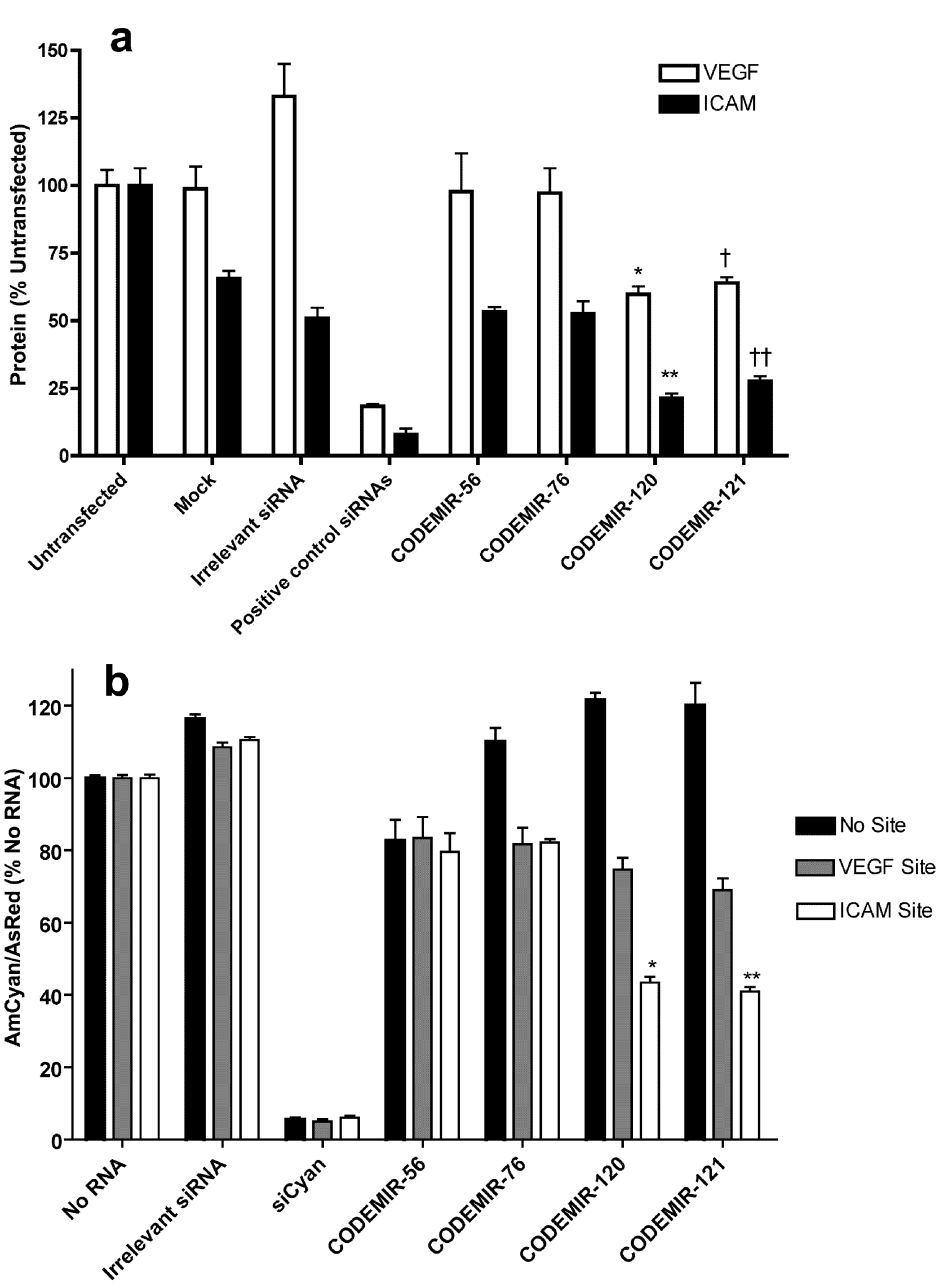
**Comparison of CODEMIR-1 variants with (#56 and #76) and without (#120 and #121) 7 G motifs**. (a) ARPE-19 cells were transfected with 40 nM duplex RNA and VEGF (ELISA) or ICAM (FACS) were assayed 48 hours post-transfection (*p < 0.001, **p < 0.01 as compared to CODEMIR-56; † p < 0.01, †† p < 0.05 as compared to CODEMIR-76). (b) Normalised fluorescence of ARPE-19 cells transfected with AmCyan/CODEMIR-1 reporter and AsRed plasmids and CODEMIR variants with and without 7 G motifs. ARPE-19 cells were co-transfected with 1 μg of the plasmids and 40 nM indicated RNA duplexes. Fluorescence was assessed by FACS 48 hours post-transfection (*p < 0.001 as compared to CODEMIR-56, **p < 0.001 as compared to CODEMIR-76). Each bar represents the mean of triplicate samples. Error bars indicate standard deviation. Statistical significance was determined by two-way ANOVA using a Bonferroni post-test.

Because the CODEMIR-1 target site appeared particularly amenable to suppression, we focused on variants of this CODEMIR. In a systematic approach, 32 variants of CODEMIR-1 were designed by developing a consensus sequence for the *VEGF-A *and *ICAM-1 *target sites and alternating between the *VEGF-A *and *ICAM-1 *complementary base at mismatched positions (Additional files 1 and 2) with the exception of mismatches that could be accommodated for by wobble base pairing with the eventual guide strand (eg G binding to either C or U). All 32 of these CODEMIRs were tested for the ability to suppress both endogenous *VEGF-A *and *ICAM-1 *(Fig. 3) and a cyan fluorescent protein reporter (Additional file 2) in cell culture. Suppression of the endogenous genes correlated well with suppression of the reporter (Additional file 2), particularly for *VEGF-A *where the range of suppression was greater. For *VEGF-A*, there was a clear positive correlation between activity and the length of the contiguous complementary region (from the 5' end of the guide strand) and a clear negative correlation between activity and the number of mismatches between the target and the CODEMIR (Additional file 2). By contrast, there was no such correlation between *ICAM-1 *suppression and the length of the contiguous stretch of complementarity and the correlation between activity and the number of mismatches between the target and the CODEMIR was, in this case, reversed (Additional file 2). Indeed, the variant (CODEMIR-64) which was completely complementary to the *ICAM-1 *mRNA exhibited very poor activity against *ICAM-1*. However, those CODEMIRs with high complementarity to the *ICAM-1 *mRNA all contained a region of at least 5 contiguous G nucleotides, a feature that is known to be detrimental to siRNA activity [8]. Substitution of the G at position 14 of the guide strand with an A in CODEMIR-56 and CODEMIR-76 (replacing the predicted G:U wobble base with an A:U base pair; Additional file 1) significantly improved the suppressive activity of these CODEMIRs against both the endogenous and reporter genes (Fig. 4a, b); confirming that runs of G nucleotides impair CODEMIR activity.

**Figure 5:**
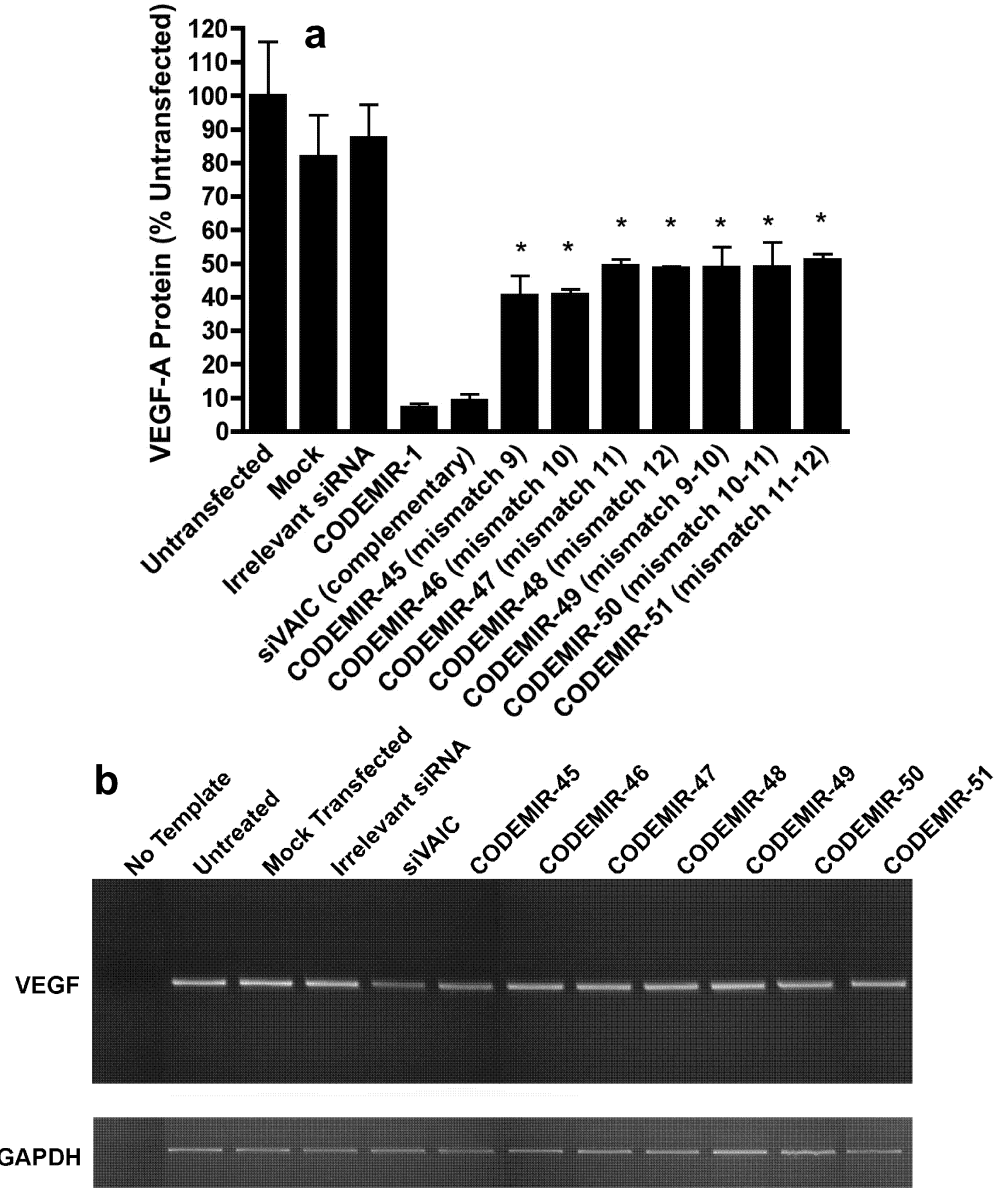
**Central mismatches at the CODEMIR-1 target site impair *VEGF-A *suppressive activity**. (a) ARPE-19 cells were transfected with 40 nM of the indicated RNA duplex, and VEGF-A secretion was measured by ELISA 48 hours post-transfection (*p < 0.001 by ANOVA as compared to CODEMIR-1). All data points represent the mean of triplicate samples. Error bars indicate standard deviation. (b) VEGF mRNA expression in ARPE-19 cells transfected with centrally mismatched variants of CODEMIR-1. Semi-quantitative RT-PCR performed on RNA from ARPE-19 cells transfected with 40 nM indicated RNA duplexes demonstrated impaired degradation of *VEGF-A *mRNA by CODEMIRs mismatched relative to a perfectly complementary control (siVAIC). Gel is representative result from duplicate experiments.

As confirmation that the length of 5' complementarity between CODEMIR-1 and the *VEGF-A *mRNA was critical to suppressive activity, we compared the activity of an interfering RNA complementary to the CODEMIR-1 *VEGF-A *target site (siVAIC) with 5 variants containing introduced mismatches at positions 9, 10, 11 and/or 12 of the guide strand (Additional file 1 and Fig. 5a). Mismatches at any of these positions reduced suppressive activity from >90% to 50–60%. This suggested that these central mismatches abrogated RISC-mediated cleavage of the mRNA, but that translational suppression, causing the retained moderate reduction in VEGF-A protein levels, was less dependent upon complementarity at these central positions. This was confirmed by semi-quantitative RT-PCR detection of the *VEGF-A *mRNA (Fig. 5b) which demonstrated reduction of *VEGF-A *transcript level with an siRNA (siVAIC) directed to the CODEMIR-1 site, but not with any of the central mismatch containing variants.

**Figure 6:**
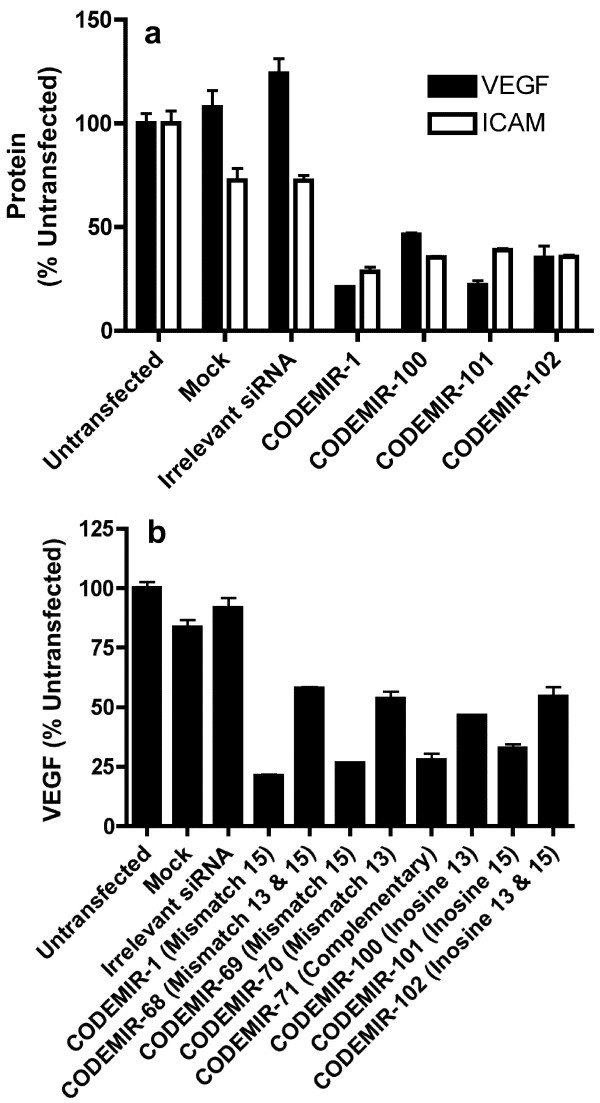
**(a) VEGF and ICAM expression in ARPE-19 cells after transfection with inosine containing CODEMIRs (#100–102)**. ARPE-19 cells were transfected with 40 nM duplex RNA and VEGF-A (ELISA) or ICAM-1 (FACS) were assayed 48 hours post-transfection. (b) Comparison of *VEGF-A *suppressive activity of CODEMIRs containing inosine bases or mismatches at positions 13 and/or 15 of the guide strand. ARPE-19 cells were transfected with 10 nM duplex RNA and VEGF-A (ELISA) was assayed 48 hours post-transfection. Each bar represents the mean of triplicate samples. Error bars indicate standard deviation.

Since the length of 5' complementarity appeared to be relevant to activity on VEGF-A, we investigated CODEMIRs which contained inosine bases (which are capable of base pairing with all 4 naturally occurring ribobases, albeit with varying affinities) at crucial points (sites that cannot be matched to all transcripts and are near the RISC cleavage site). Three variants of CODEMIR-1 were designed which included inosine bases at positions 13 and/or 15 of the guide strand (CODEMIRs 100–102; Additional file 1). These CODEMIRs showed comparable *ICAM-1 *suppressive activity to CODEMIR-1 (which contains a mismatch at position 13), but reduced *VEGF-A *suppression relative to CODEMIR-1 in the case of CODEMIR-100 and CODEMIR-102 (Fig. 6a). The comparable activity against *ICAM-1 *may simply reflect the fact that translational repression against *ICAM-1 *is largely dependent upon the seed binding alone, and so is not affected by alterations in the 3' tail. The *VEGF-A *suppressive activity of inosine-containing variants of CODEMIR-1 was also compared to similar variants of CODEMIR-1 with mismatches to the *VEGF-A *mRNA at positions 13 and/or 15 (CODEMIRs 68–71; Additional file 1). None of the inosine-containing variants demonstrated substantially improved activity compared to the corresponding mismatched variant (Fig. 6b). This indicates that inosine base pairs, although tolerated in some positions, may not necessarily be useful in overcoming mismatches between guide strand and target, although our analysis may not be generalizable given that the binding of dI to each base varies and is context-dependent.

**Figure 7:**
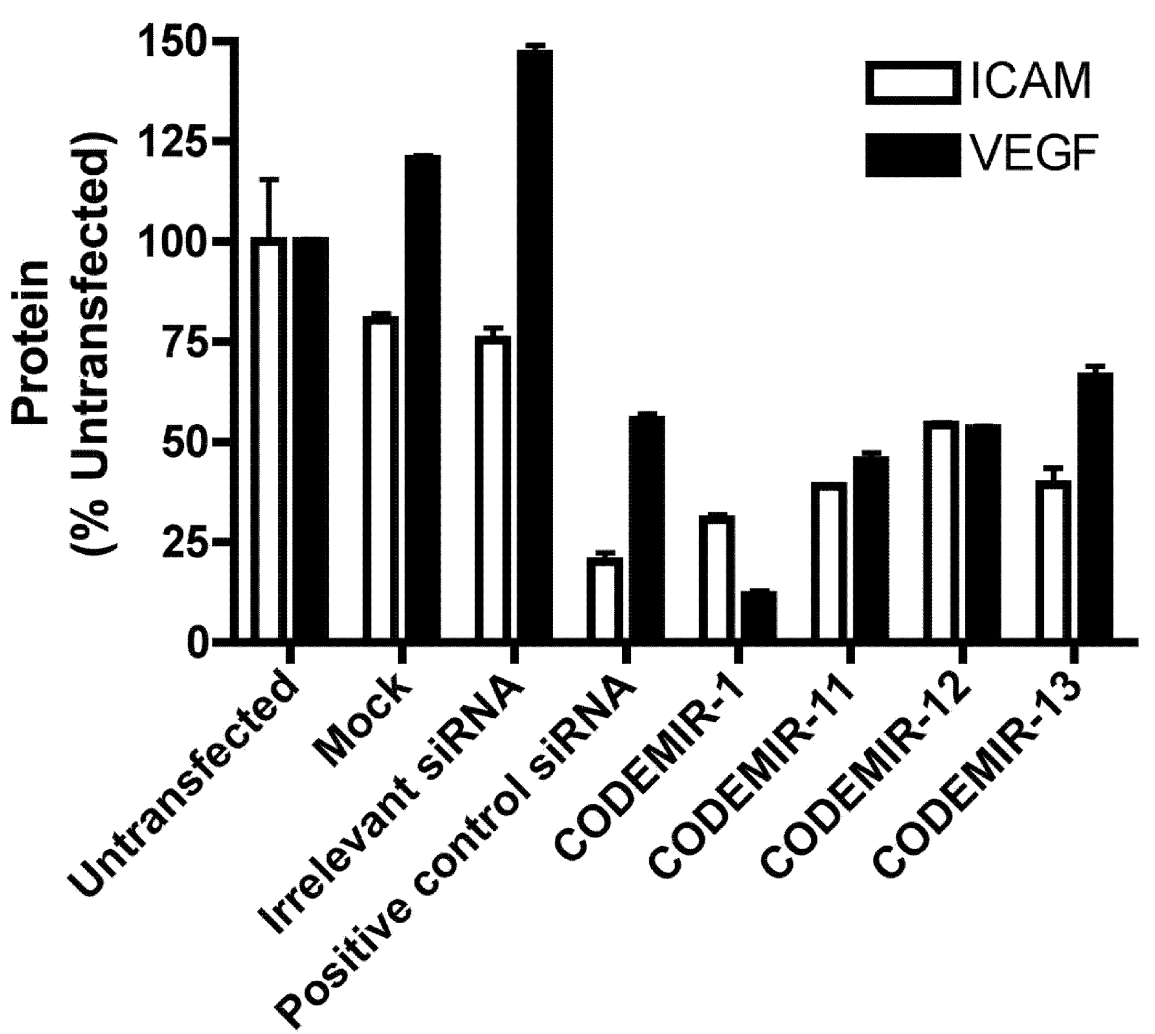
**Suppression of *VEGF-A *and *ICAM-1 *expression by variants (shifted 5' of the guide strand) of CODEMIR-1**. ARPE-19 cells were transfected with 40 nM of the indicated duplex RNA. 24 hours after transfection cells were stimulated with 130 μM Deferoxamine (*VEGF-A*) or 1 ng/ml IL-1β (*ICAM-1*). Gene expression was assayed 48 hours after transfection by ELISA on cell supernatant (VEGF-A) or FACS (ICAM). All data points indicate the mean of triplicate samples. Error bars indicate standard deviation.

There are 3 bases common to both *ICAM-1 *and *VEGF-A *immediately downstream (3') of the CODEMIR-1 target site. We investigated whether increasing the length of the complementary region (to both targets) would increase activity. Three variants of CODEMIR-1 were designed such that the guide strand sequence was shifted 1–3 bases 5' of the CODEMIR-1 sequence (Additional file 1). However, none of these were as active as CODEMIR-1 against either target (Fig. 7). Whilst the variants shifted 1 and 2 bases (CODEMIRs 11 and 12) would be predicted to have compromised strand loading due to the introduction of a G:C base pair close to the 5' end of the guide strand, the variant shifted 3 bases (CODEMIR-13) would be predicted to retain a strong loading bias towards the guide strand.

**Figure 8:**
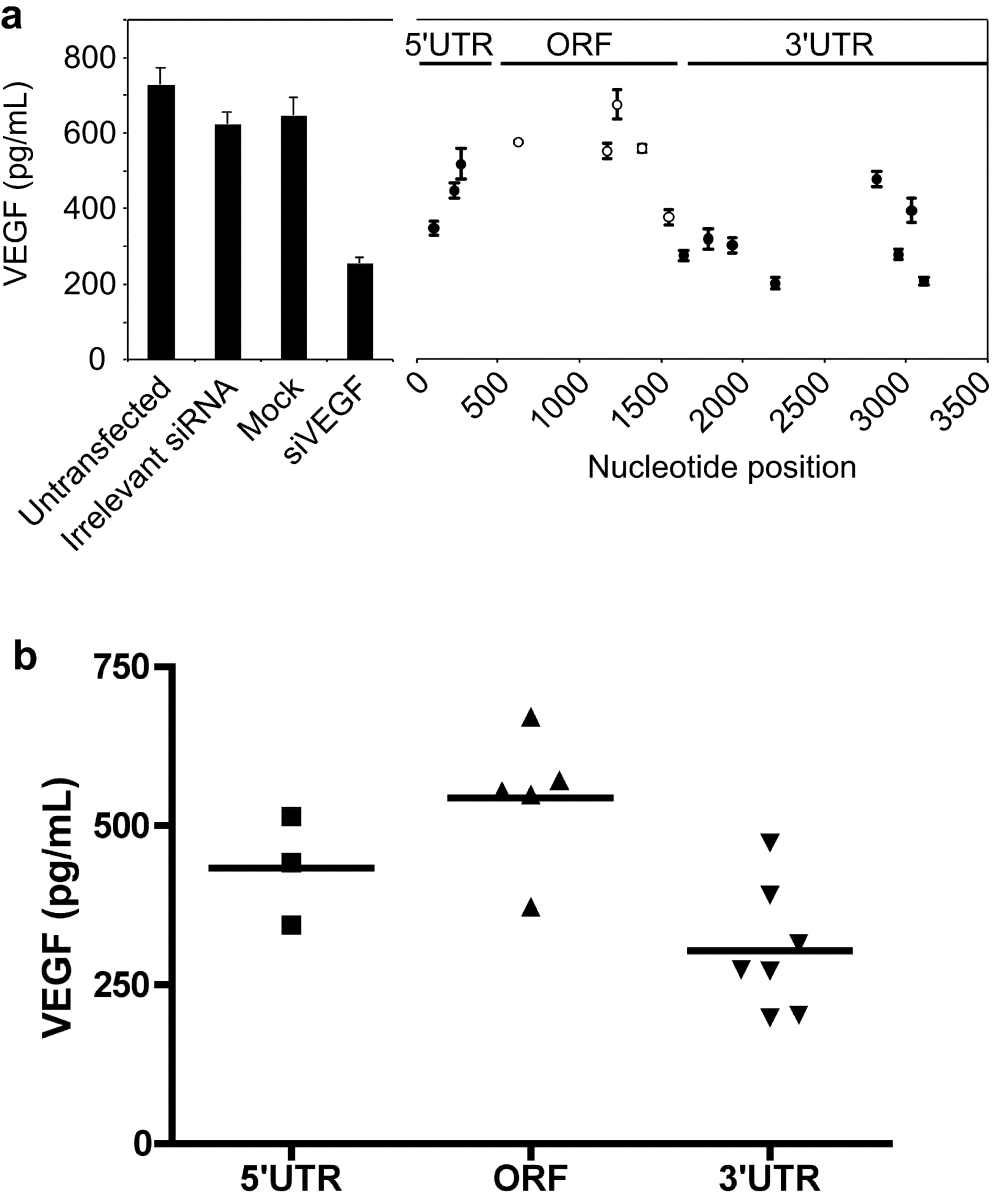
**Analysis of target site selection**. (a) *VEGF-A *suppression by 21 nt RNA duplexes (with central mismatches) targeting different regions of the *VEGF-A *mRNA. ARPE-19 cells were transfected with 40 nM indicated RNA duplexes. VEGF-A in cell culture supernatant was measured by ELISA 48 hours post-transfection and 24 hours post-stimulation with 130 μM Deferoxamine. Each point represents the mean of triplicate samples. Error bars indicate standard deviation. (b) Statistical analysis of data for *VEGF-A *suppression depicted in Panel (a). There was a significant difference (p < 0.05) between the means for molecules targeting the 3'UTR as compared to molecules targeting the ORF as determined by one-way ANOVA with a Bonferroni post-test.

Mammalian miRNAs have generally been shown to bind in the 3' UTRs of target genes, and the CODEMIRs described above all targeted 3' UTRs. To assess whether the targeting of 3' UTR regions is advantageous when there is incomplete complementarity between the target and the guide strand of a duplex RNA in the central region (known to be required for Ago-2 cleavage in the case of siRNA) dsRNA duplexes were designed such that they were completely complementary to the *VEGF-A *mRNA, excepting the nucleotides at positions 10 and 11 of the guide strand (Additional file 1). To minimize variations in efficacy due to sequence composition or strand-loading bias, each of these duplexes contained a similar bulge composition (CU) and was relatively GC rich at the 3' end of the guide strand. Of these 16, 3 targeted the *VEGF-A *5' UTR, 5 targeted the *VEGF-A *ORF and 8 targeted the *VEGF-A *3'UTR. In general, the duplexes targeting regions in the 5' UTR or ORF displayed poor *VEGF-A *suppression, whereas those targeting regions in the 3' UTR displayed strong suppression (Fig. 8a). Moreover, there was a significant difference in activity between the duplexes targeting the ORF and those targeting the 3'UTR (p < 0.01; Fig. 8b). This demonstrates that the 3' UTR of *VEGF-A *mRNA is, relative to other regions of the mRNA, more suited to suppression through miRNA-like translational repression, which in part is likely to account for the high activity of CODEMIR-1 and its variants against this target.

The use of RNA duplexes is potentially confounded by off-target inflammatory cellular responses [16]. To confirm the specificity of action of CODEMIR-1 we examined the expression of *IFNβ *and *STAT1*. After transfection with CODEMIR-1, no evidence of up-regulation of either of these genes was observed (Additional file 2). In a recent separate study to be reported elsewhere, we studied the activation of TLR7/8 by 207 siRNA sequences when transfected with DOTAP into fresh human PBMCs at concentrations up to 100 nM. CODEMIR-1 belonged to the least active subset of sequences (with IC_50 _> 100 nM), confirming low propensity of this sequence for activation of RNA-sensing innate receptors.

## Discussion

The present study demonstrates proof of concept for the approach of using short interfering RNAs with at least partial complementarity to two target transcripts for suppression of the expression of unrelated genes. Although we have only presented data for two seed sequences in two genes here, we have successfully used the same approach to target unrelated genes implicated in diverse disease states including oncology, virology and inflammation [17]. Thus, this approach can be used as a general technique for the suppression of multiple genes using a single interfering RNA.

Current pharmaceutical research is dominated by a reductionist "one-disease one-target" paradigm. However, the complex nature of many diseases has increasingly led to the realisation that activity against multiple targets may be required for effective treatment [18]. Practically, this can be achieved through the use of multiple agents, and a number of combination drugs have recently become available for the treatment of heart disease and HIV infection, amongst other indications. However, the combination of multiple agents can lead to unforseen interactions [19]. Alternatively, agents with activity against multiple targets can be developed [20]. Indeed, many successful drugs in different areas of medicine (eg clozapine, imatinib) are active precisely because of their promiscuity of action [21,22]. To date, the RNAi-based drugs that have been investigated have been designed for single specific targets, with efforts taken to reduce non-specific effects [23,24]. However, in some instances, the processes they target are distinctly polygenic (eg cholesterol metabolism and angiogenesis), and the targeting of multiple genes seems likely to be therapeutically beneficial. Whilst the targeting of multiple genes could be achieved through the use of a mixture of active siRNAs, we believe that using a single active has a number of possible advantages. First, having a single active reduces the complexity of clinical and product development. Secondly, mixtures of siRNA can have disappointing effects because of competition for the RISC machinery [25]. Thirdly, a single active may have reduced off-target effects relative to a pool of actives, since each siRNA has a unique pattern of off-target effects and a mixture may thus increase the magnitude and/or scope of off-target effects.

Our finding that the length of seed complementarity affects CODEMIR activity independent of overall complementarity is surprising given the current understanding of microRNA-target interactions [4,9,11]. Mismatches to the target in the central positions of the CODEMIR-1 guide strand decreased *VEGF-A *suppression at the protein level to 50–60%, and largely abrogated suppression at the RNA level. This suggests that complementarity to the target at the 5'-end and in the centre of the guide strand is sufficient to induce target cleavage, and that CODEMIRs with central mismatches to the targets may act primarily through translational repression. As such, CODEMIRs with longer 5' regions of contiguous complementarity may exhibit higher efficacy. However, suppression of multiple genes related to the same phenotype is likely to have significant advantages over targeting a single gene, and thus CODEMIRs with central mismatches will still be of significant utility.

## Conclusion

The present study validates the approach of using a short dsRNA molecule specifically designed with a single guide strand to suppress the expression of multiple unrelated genes implicated in a particular medical condition. We have shown that synthetic duplex RNAs with at least partial complementarity to multiple transcripts are capable of specific suppression of multiple target genes. Given the multi-genic nature of many disease states, such multi-targeting interfering RNAs may offer significant therapeutic benefits relative to single target siRNAs. Our findings also have relevance to the biology of miRNA-target interactions, particularly with respect to the effect of unpaired bases on miRNA-mediated suppression of translation.

## Methods

### Cell culture and transfection

ARPE-19 cells were cultured in Dulbecco's Modified Eagle's Medium supplemented with 10% Fetal bovine serum and 10 mM glutamine (Gibco). Duplex RNA transfection was performed using Lipofectamine 2000 (Invitrogen), at a ratio of 260 ng siRNA per 1 μL Lipofectamine, according to the manufacturer's instructions. Cells were transfected 24 hours after seeding at a density of 1.25 – 10^4 ^cells per cm^2 ^in 96, 48 or 24-well plates. RNA duplex and plasmid co-transfection was performed with 1 μg reporter plasmid (AmCyan based – see below), 1 μg control plasmid (pAsRed-C1 – Clontech) and 40 pmol RNA duplex together complexed with 10 μg Lipofectamine 2000 and applied to cells in a 12 well plate.

### ELISA and FACS analysis of gene expression

VEGF-A concentrations in cell supernatants were assayed 48 hours after transfection and 24 hours after stimulation with 130 μM Deferoxamine (Sigma) using a commercially available ELISA kit (R&D Systems) according to the manufacturer's instructions. Cell surface ICAM-1 was assayed by flow cytometry. Cells seeded into 12 well plates were transfected with RNA duplexes 24 hours after seeding. *ICAM-1 *expression was assayed 48 hours after transfection and 24 hours after stimulation with 1 ng/mL recombinant human Interleukin-1β (R&D systems): cells were trypsinized, stained with 0.5 μg anti-human ICAM-1 mouse IgG_1 _antibody (Becton Dickinson) at 4°C for 20 minutes, washed with PBS, stained with 0.2 μg Phycoerythrin-labelled anti-mouse IgG_1 _antibody (Becton Dickinson) at 4°C for 20 minutes, washed with PBS and analysed using a FACScalibur flow cytometer (Becton Dickinson). IFNβ production was assayed using a commercially available ELISA (InVitrogen) according to the manufacturer's instructions. For detection of fluorescent reporter expression, mean fluorescence in the reporter channel (FL-1) was normalised to mean fluorescence in the control channel (FL-2). Statistical analysis was performed using Prism software (GraphPad Software Inc.)

### RNA quantification

For semi-quantitative RT-PCR, total cellular RNA was extracted using the RNeasy kit (Qiagen) according to the manufacturer's instructions. Reverse transcription was performed on 1 μg total RNA using a commercially available kit (First-Strand cDNA Synthesis Kit, Marligen Biosciences). PCR of GAPDH was used to standardise the amounts of starting cDNA. PCR was performed in 1× PCR buffer II (Applied Biosystems) supplemented with 3.75 mM MgCl_2_, 250 μM each dNTP, 250 nM each primer and 0.125 U/μL AmpliTaq gold (Applied Biosystems). *VEGF-A *was amplified by 30 cycles of 95°C – 30 seconds, 60°C – 30 seconds and 72°C – 30 seconds, using the primers (5' to 3'): TTC TTG CTG CTA AAT CAC CGA and GAA CAT TCC CCT CCC AAC TCA. GAPDH was amplified by 18 cycles of 95°C – 30 seconds, 65°C – 30 seconds and 72°C – 30 seconds, using the primers (5' to 3'): CTG CTT CAC CAC CTT CTT GAT GTC ATC ATA and GAC CCC TTC ATT GAC CTC AAC TAC ATG GT.

*VEGF-A*, *ICAM-1*, *IFN β*, *STAT1 *and *GAPDH *RNA were quantified using a Quantigene^® ^branched DNA assay (Panomics), according to the manufacturer's instructions.

### Plasmid construction

Fluorescent reporter vectors were constructed by cloning target sites into the 3'UTR of the AmCyan1 fluorescent protein gene in the pAmCyan1-C1 vector (Clontech). A stop codon was inserted by cloning the duplex oligodeoxynucleotide pair GAT CTC TCG AGT GAT AGG and AAT TCC TAT CAC TCG AGA into the *Bgl*II and *Eco*RI sites of pAmCyan1-C1. Specific CODEMIR-1 target site reporters were generated by cloning the duplex oligonucleotide pairs AAT TTC CTG TAG ACA CAC CCA CCC ACA TAC and GAT CGT ATG TGG GTG GGT GTG TCT ACA GGA (VEGF-A), and AAT TTG TTA GCC ACC TCC CCA CCC ACA TAC and GAT CGT ATG TGG GTG GGG AGG TGG CTA ACA (ICAM-1) into the *Eco*RI and *Bam*HI sites of the stop codon-containing pAmCyan1-C1 vector. The full-length VEGF-A 3'UTR (including stop codon) was cloned from ARPE-19 cells by RT-PCR using the primers GGG CTC GAG TGA GCC GGG CAG GAG G (Forward) and GGG GTC GAC TAC GGA ATA TCT CGA AAA ACT (Reverse) and cloned into the *Xho*I and *Sal*I sites of the pAmCyan1-C1.

### Western blot analysis

Total protein lysates were obtained by lysing cells in RIPA buffer (150 mM NaCl, 0.1% sodium dodecyl sulphate, 1% nonidet P-40, 0.5% sodium deoxycholate, 50 mM Tris-Cl, pH 8). Protein concentrations were determined by the Lowry protocol using the Bio-Rad *D*_*C *_Protein Assay. Total proteins (10 μg) were separated by electrophoresis on NuPage 4–12% Bis-Tris gels (Invitrogen) and transferred onto nitrocellulose membranes. Membranes were blocked with 3% BSA in TBST (10 mM Tris pH 8, 30 mM NaCl, 0.05% Tween) for 20 min at room temperature. After rinsing with TBST twice, anti-STAT1 mouse IgG_1 _(1:400; Santa Cruz Biotechnology) and anti-β-actin mouse IgG_1 _(1:2000; Sigma-Aldrich) in TBST-MLK (TBST containing 5% dried skim milk) were added and incubated for 1 hour at room temperature. After 3 – 5 min washes in TBST, membranes were incubated with horseradish peroxidase conjugated anti-mouse Ig (1:2000 in TBS-MLK; Dako Cytomation) for 45 min at room temperature. The membrane was washed in TBST (3 – 5 min) and analysed by chemiluminescence using ECL Western blotting detection (Amersham).

## Authors' contributions

TP performed *in vitro *cell assays, ELISAs, FACS analysis, molecular cloning and drafted the manuscript. MMG carried out the immune stimulation assays. AG performed *in vitro *cell assays and ELISAs. AK performed the RT-PCR. MP performed the bioinformatic analyses. GMA and DJB conceived of, and participated in the design of the study. LPR conceived of, and participated in the design of the study and helped draft the manuscript. All authors read and approved the final manuscript.

## Supplementary Material

Additional file 1 **Additional tables**. Tabulated sequence data for the nucleic acids used in the study. Click here for file

Additional file 2 **Additional figures**. Figures and legends demonstrating supporting data for the study. Click here for file

## References

[B1] Schwarz DS, Ding H, Kennington L, Moore JT, Schelter J, Burchard J, Linsley PS, Aronin N, Xu Z, Zamore PD (2006). Designing siRNA That Distinguish between Genes That Differ by a Single Nucleotide. PLoS Genet.

[B2] Kloosterman WP, Plasterk RH (2006). The diverse functions of microRNAs in animal development and disease. Dev Cell.

[B3] Khvorova A, Reynolds A, Jayasena SD (2003). Functional siRNAs and miRNAs exhibit strand bias. Cell.

[B4] Engels BM, Hutvagner G (2006). Principles and effects of microRNA-mediated post-transcriptional gene regulation. Oncogene.

[B5] Filipowicz W, Jaskiewicz L, Kolb FA, Pillai RS (2005). Post-transcriptional gene silencing by siRNAs and miRNAs. Curr Opin Struct Biol.

[B6] Schwarz DS, Hutvagner G, Du T, Xu Z, Aronin N, Zamore PD (2003). Asymmetry in the assembly of the RNAi enzyme complex. Cell.

[B7] Martinez J, Patkaniowska A, Urlaub H, Luhrmann R, Tuschl T (2002). Single-stranded antisense siRNAs guide target RNA cleavage in RNAi. Cell.

[B8] Reynolds A, Leake D, Boese Q, Scaringe S, Marshall WS, Khvorova A (2004). Rational siRNA design for RNA interference. Nat Biotechnol.

[B9] Brennecke J, Stark A, Russell RB, Cohen SM (2005). Principles of microRNA-target recognition. PLoS Biol.

[B10] Doench JG, Sharp PA (2004). Specificity of microRNA target selection in translational repression. Genes Dev.

[B11] Grimson A, Farh KK, Johnston WK, Garrett-Engele P, Lim LP, Bartel DP (2007). MicroRNA targeting specificity in mammals: determinants beyond seed pairing. Mol Cell.

[B12] Lai EC (2002). Micro RNAs are complementary to 3' UTR sequence motifs that mediate negative post-transcriptional regulation. Nat Genet.

[B13] Funatsu H, Yamashita H, Sakata K, Noma H, Mimura T, Suzuki M, Eguchi S, Hori S (2005). Vitreous levels of vascular endothelial growth factor and intercellular adhesion molecule 1 are related to diabetic macular edema. Ophthalmology.

[B14] Hofacker IL (2003). Vienna RNA secondary structure server. Nucleic Acids Res.

[B15] Rehmsmeier M, Steffen P, Hochsmann M, Giegerich R (2004). Fast and effective prediction of microRNA/target duplexes. Rna.

[B16] Marques JT, Devosse T, Wang D, Zamanian-Daryoush M, Serbinowski P, Hartmann R, Fujita T, Behlke MA, Williams BR (2006). A structural basis for discriminating between self and nonself double-stranded RNAs in mammalian cells. Nat Biotechnol.

[B17] Rivory LRPM, Birkett DJ, Arndt GM, Passioura TJ Multitargeting Interfering RNAs and Methods of their Use and Design.

[B18] Imming P, Sinning C, Meyer A (2006). Drugs, their targets and the nature and number of drug targets. Nat Rev Drug Discov.

[B19] Frantz S (2006). The trouble with making combination drugs. Nat Rev Drug Discov.

[B20] Morphy R, Rankovic Z (2006). The physicochemical challenges of designing multiple ligands. J Med Chem.

[B21] Meltzer HY (1994). An overview of the mechanism of action of clozapine. J Clin Psychiatry.

[B22] Pardanani A, Tefferi A (2004). Imatinib targets other than bcr/abl and their clinical relevance in myeloid disorders. Blood.

[B23] Jackson AL, Burchard J, Schelter J, Chau BN, Cleary M, Lim L, Linsley PS (2006). Widespread siRNA "off-target" transcript silencing mediated by seed region sequence complementarity. Rna.

[B24] Zimmermann TS, Lee AC, Akinc A, Bramlage B, Bumcrot D, Fedoruk MN, Harborth J, Heyes JA, Jeffs LB, John M (2006). RNAi-mediated gene silencing in non-human primates. Nature.

[B25] Castanotto D, Sakurai K, Lingeman R, Li H, Shively L, Aagaard L, Soifer H, Gatignol A, Riggs A, Rossi JJ (2007). Combinatorial delivery of small interfering RNAs reduces RNAi efficacy by selective incorporation into RISC. Nucleic Acids Res.

