# Supplementary material for: Interfering ribonucleic acids that suppress expression of multiple unrelated genes
Source: BMC Biotechnol. 2009 Jun 16;9:57. doi: 10.1186/1472-6750-9-57 (PMC2706242; doi:10.1186/1472-6750-9-57)
Supplement: Additional file 1 — Additional tables. Tabulated sequence data for the nucleic acids used in the study. [file 1472-6750-9-57-S1.doc]

Supplementary Table 1 – Control siRNA sequences

|  | Guide strand (5’ to 3’) |
| --- | --- |
| Irrelevant control siRNA | GUCUGCGAUCGCAUACAAUdTdT |
| VEGF-A siRNA | GUGCUGGCCUUGGUGAGGUdTdT |
| ICAM-1 siRNA | UAGAGGUACGUGCUGAGGCdTdT |
| Cyan Fluorescent Protein siRNA | UUCACGGUGAAGUAGUGGCdTdT |

Supplementary Table 2 – Variants of CODEMIR-1 with seed mismatches.

|  | Duplex | mRNA binding (RNA hybrid) |
| --- | --- | --- |
| CODEMIR-1 | Passenger  AGACUCACCCACCCACAUAUU  AAUCUGAGUGGGUGGGUGUAU  Guide | VEGF 5' G A C 3'  UAGAC CACCCACCCACAUA  AUCUG GUGGGUGGGUGUAU  3' A A 5' |
|  |  | ICAM 5' G CCAC C 3'  UUAG CUC CCCACCCACAUA  AAUC GAG GGGUGGGUGUAU  3' U U 5' |
| CODEMIR-122 | Passenger  AGACUCACCCACCCAGAUAUU  AAUCUGAGUGGGUGGGUCUAU  Guide | VEGF 5' G A C C 3'  UAGAC CACCCACCCA AUA  AUCUG GUGGGUGGGU UAU  3' A A C 5' |
|  |  | ICAM 5' G CCAC C C 3'  UUAG CUC CCCACCCA AUA  AAUC GAG GGGUGGGU UAU  3' U U C 5' |
| CODEMIR-123 | Passenger  AGACUCACCCACCGAGAUAUU  AAUCUGAGUGGGUGGCUCUAU  Guide | VEGF 5' G A CACAUAC 3'  UAGAC CACCCACC  AUCUG GUGGGUGG  3' A A CUCUAU 5' |
|  |  | ICAM 5' G CCAC CACAUA 3'  UUAG CUC CCCACC  AAUC GAG GGGUGG  3' U U CUCUAU 5' |
| CODEMIR-124 | Passenger  AGACUCACCCAGCGAGAUAUU  AAUCUGAGUGGGUCGCUCUAU  Guide | VEGF 5' G A CCCACAUAC 3'  UAGAC CACCCA  AUCUG GUGGGU  3' A A CGCUCUAU 5' |
|  |  | ICAM 5' G CCAC CCCACAUA 3'  UUAG CUC CCCA  AAUC GAG GGGU  3' U U CGCUCUAU 5' |

**Supplementary Table 3 – 3’ variants of CODEMIR-1**

|  | **Guide strand (5’ to 3’)** | **VEGF binding (upper = VEGF mRNA)** | **ICAM-1 binding (upper = ICAM-1 mRNA)** |
| --- | --- | --- | --- |
| CODEMIR-1 | UAUGUGGGUGGGUGAGUCUAA | 5' G A C 3'  UAGAC CACCCACCCACAUA  AUCUG GUGGGUGGGUGUAU  3' A A 5' | 5' G CCAC C 3'  UUAG CUC CCCACCCACAUA  AAUC GAG GGGUGGGUGUAU  3' U U 5' |
| CODEMIR-52 | UAUGUGGGUGGGGGGGUCUCU | 5' U UGUAGACACA C 3'  GGGAUUCC CCCACCCACAUA  CUCUGGGG GGGUGGGUGUAU  3' U 5' | 5' U CC C 3'  AG ACCUCCCCACCCACAUA  UC UGGGGGGGUGGGUGUAU  3' UC 5' |
| CODEMIR-53 | UAUGUGGGUGGGUGGGUCUCU | 5' U A C 3'  AGAC CACCCACCCACAUA  UCUG GUGGGUGGGUGUAU  3' UC G 5' | 5' U CC C C 3'  AG ACCU CCCACCCACAUA  UC UGGG GGGUGGGUGUAU  3' UC U 5' |
| CODEMIR-54 | UAUGUGGGUGGGGGUGUCUCU | 5' U A C 3'  AGACAC CCCACCCACAUA  UCUGUG GGGUGGGUGUAU  3' UC G 5' | 5' U CUUUGUUAGCCACCU C 3'  GACAC CCCCACCCACAUA  CUGUG GGGGUGGGUGUAU  3' UCU 5' |
| CODEMIR-55 | UAUGUGGGUGGGUGUGUCUCU | 5' U C 3'  AGACACACCCACCCACAUA  UCUGUGUGGGUGGGUGUAU  3' UC 5' | 5' U C CUC C 3'  AG CAC CCCACCCACAUA  UC GUG GGGUGGGUGUAU  3' UC U U 5' |
| CODEMIR-56 | UAUGUGGGUGGGGGGGUGUCU | 5' U A C 3'  AGACAC CCCACCCACAUA  UCUGUG GGGUGGGUGUAU  3' GGG 5' | 5' U C C 3'  AG CACCUCCCCACCCACAUA  UC GUGGGGGGGUGGGUGUAU  3' U 5' |
| CODEMIR-57 | UAUGUGGGUGGGUGGGUGUCU | 5' U C 3'  AGACAC ACCCACCCACAUA  UCUGUG UGGGUGGGUGUAU  3' GG 5' | 5' U C C C 3'  AG CACCU CCCACCCACAUA  UC GUGGG GGGUGGGUGUAU  3' U U 5' |
| CODEMIR-58 | UAUGUGGGUGGGGGUGUGUCU | 5' U C 3'  AGACACA CCCACCCACAUA  UCUGUGU GGGUGGGUGUAU  3' GG 5' | 5' U C C C 3'  AG CAC UCCCCACCCACAUA  UC GUG GGGGGUGGGUGUAU  3' U U 5' |
| CODEMIR-59 | UAUGUGGGUGGGUGUGUGUCU | 5' G UCCUGUAG C 3'  GGAU ACACACCCACCCACAUA  UCUG UGUGUGGGUGGGUGUAU  3' 5' | 5' U CUUUGUUAGCC CUC C 3'  GACAC AC CCCACCCACAUA  CUGUG UG GGGUGGGUGUAU  3' U U 5' |
| CODEMIR-60 | UAUGUGGGUGGGGGGGUCGCU | 5' U G UGUAGACACA C 3'  G GAUUCC CCCACCCACAUA  C CUGGGG GGGUGGGUGUAU  3' U G 5' | 5' U C C 3'  AGC ACCUCCCCACCCACAUA  UCG UGGGGGGGUGGGUGUAU  3' C 5' |
| CODEMIR-61 | UAUGUGGGUGGGUGGGUCGCU | 5' A A C 3'  GAC CACCCACCCACAUA  CUG GUGGGUGGGUGUAU  3' UCG G 5' | 5' U C C C 3'  AGC ACCU CCCACCCACAUA  UCG UGGG GGGUGGGUGUAU  3' C U 5' |
| CODEMIR-62 | UAUGUGGGUGGGGGUGUCGCU | 5' A A C 3'  GACAC CCCACCCACAUA  CUGUG GGGUGGGUGUAU  3' UCG G 5' | 5' U C C C 3'  AGC AC UCCCCACCCACAUA  UCG UG GGGGGUGGGUGUAU  3' C U 5' |
| CODEMIR-63 | UAUGUGGGUGGGUGUGUCGCU | 5' A C 3'  GACACACCCACCCACAUA  CUGUGUGGGUGGGUGUAU  3' UCG 5' | 5' U C UC C 3'  AGC AC C CCCACCCACAUA  UCG UG G GGGUGGGUGUAU  3' C U U 5' |
| CODEMIR-64 | UAUGUGGGUGGGGGGGUGGCU | 5' U A A C 3'  AG CAC CCCACCCACAUA  UC GUG GGGUGGGUGUAU  3' G GGG 5' | 5' U C 3'  AGCCACCUCCCCACCCACAUA  UCGGUGGGGGGGUGGGUGUAU  3' 5' |
| CODEMIR-65 | UAUGUGGGUGGGUGGGUGGCU | 5' U UG AGACA C 3'  CC U CACCCACCCACAUA  GG G GUGGGUGGGUGUAU  3' UC UG 5' | 5' U C C 3'  AGCCACCU CCCACCCACAUA  UCGGUGGG GGGUGGGUGUAU  3' U 5' |
| CODEMIR-66 | UAUGUGGGUGGGGGUGUGGCU | 5' U AG A C 3'  GU ACAC CCCACCCACAUA  CG UGUG GGGUGGGUGUAU  3' U G G 5' | 5' U C C 3'  AGCCAC UCCCCACCCACAUA  UCGGUG GGGGGUGGGUGUAU  3' U 5' |
| CODEMIR-67 | UAUGUGGGUGGGUGUGUGGCU | 5' U AG C 3'  GU ACACACCCACCCACAUA  CG UGUGUGGGUGGGUGUAU  3' U G 5' | 5' U UC C 3'  AGCCAC C CCCACCCACAUA  UCGGUG G GGGUGGGUGUAU  3' U U 5' |
| CODEMIR-68 | UAUGUGGGUGGGGGGGUCUAU | 5' U ACA C 3'  GUAGAC CCCACCCACAUA  UAUCUG GGGUGGGUGUAU  3' GGG 5' | 5' U CC C 3'  UAG ACCUCCCCACCCACAUA  AUC UGGGGGGGUGGGUGUAU  3' U 5' |
| CODEMIR-69 | UAUGUGGGUGGGUGGGUCUAU | 5' U A C 3'  GUAGAC CACCCACCCACAUA  UAUCUG GUGGGUGGGUGUAU  3' G 5' | 5' U CC C C 3'  UAG ACCU CCCACCCACAUA  AUC UGGG GGGUGGGUGUAU  3' U U 5' |
| CODEMIR-70 | UAUGUGGGUGGGGGUGUCUAU | 5' U A C 3'  GUAGACAC CCCACCCACAUA  UAUCUGUG GGGUGGGUGUAU  3' G 5' | 5' U C CU C 3'  UAG CAC CCCCACCCACAUA  AUC GUG GGGGUGGGUGUAU  3' U U 5' |
| CODEMIR-71 | UAUGUGGGUGGGUGUGUCUAU | 5' U C 3'  GUAGACACACCCACCCACAUA  UAUCUGUGUGGGUGGGUGUAU  3' 5' | 5' U C CUC C 3'  UAG CAC CCCACCCACAUA  AUC GUG GGGUGGGUGUAU  3' U U U 5' |
| CODEMIR-72 | UAUGUGGGUGGGGGGGUGUAU | 5' G A C 3'  ACAC CCCACCCACAUA  UGUG GGGUGGGUGUAU  3' UA GGG 5' | 5' C C 3'  CACCUCCCCACCCACAUA  GUGGGGGGGUGGGUGUAU  3' UAU 5' |
| CODEMIR-73 | UAUGUGGGUGGGUGGGUGUAU | 5' U G A C 3'  GUA AC CACCCACCCACAUA  UAU UG GUGGGUGGGUGUAU  3' G G 5' | 5' C C C 3'  CACCU CCCACCCACAUA  GUGGG GGGUGGGUGUAU  3' UAU U 5' |
| CODEMIR-74 | UAUGUGGGUGGGGGUGUGUAU | 5' U G A C 3'  GUA ACAC CCCACCCACAUA  UAU UGUG GGGUGGGUGUAU  3' G G 5' | 5' C C C 3'  CAC UCCCCACCCACAUA  GUG GGGGGUGGGUGUAU  3' UAU U 5' |
| CODEMIR-75 | UAUGUGGGUGGGUGUGUGUAU | 5' U G C 3'  GUA ACACACCCACCCACAUA  UAU UGUGUGGGUGGGUGUAU  3' G 5' | 5' A CUC C 3'  GC CAC CCCACCCACAUA  UG GUG GGGUGGGUGUAU  3' UA U U 5' |
| CODEMIR-76 | UAUGUGGGUGGGGGGGUCGAU | 5' A ACA C 3'  GAC CCCACCCACAUA  CUG GGGUGGGUGUAU  3' UAG GGG 5' | 5' C C 3'  ACCUCCCCACCCACAUA  UGGGGGGGUGGGUGUAU  3' UAGC 5' |
| CODEMIR-77 | UAUGUGGGUGGGUGGGUCGAU | 5' A A C 3'  GAC CACCCACCCACAUA  CUG GUGGGUGGGUGUAU  3' UAG G 5' | 5' C C C 3'  ACCU CCCACCCACAUA  UGGG GGGUGGGUGUAU  3' UAGC U 5' |
| CODEMIR-78 | UAUGUGGGUGGGGGUGUCGAU | 5' A A C 3'  GACAC CCCACCCACAUA  CUGUG GGGUGGGUGUAU  3' UAG G 5' | 5' C CUUUGUUAGCCACCU C 3'  UGACAC CCCCACCCACAUA  GCUGUG GGGGUGGGUGUAU  3' UA 5' |
| CODEMIR-79 | UAUGUGGGUGGGUGUGUCGAU | 5' A C 3'  GACACACCCACCCACAUA  CUGUGUGGGUGGGUGUAU  3' UAG 5' | 5' A C CUC C 3'  G CAC CCCACCCACAUA  C GUG GGGUGGGUGUAU  3' UAG U U 5' |
| CODEMIR-80 | UAUGUGGGUGGGGGGGUGGAU | 5' U UG AGACA A C 3'  UCC U C CCCACCCACAUA  AGG G G GGGUGGGUGUAU  3' U UG G 5' | 5' G C 3'  CCACCUCCCCACCCACAUA  GGUGGGGGGGUGGGUGUAU  3' UA 5' |
| CODEMIR-81 | UAUGUGGGUGGGUGGGUGGAU | 5' U UG AGACA C 3'  UCC U CACCCACCCACAUA  AGG G GUGGGUGGGUGUAU  3' U UG 5' | 5' G C C 3'  CCACCU CCCACCCACAUA  GGUGGG GGGUGGGUGUAU  3' UA U 5' |
| CODEMIR-82 | UAUGUGGGUGGGGGUGUGGAU | 5' U UGUAG A C 3'  UCC ACAC CCCACCCACAUA  AGG UGUG GGGUGGGUGUAU  3' U G 5' | 5' G C C 3'  CCAC UCCCCACCCACAUA  GGUG GGGGGUGGGUGUAU  3' UA U 5' |
| CODEMIR-83 | UAUGUGGGUGGGUGUGUGGAU | 5' U UGUAG C 3'  UCC ACACACCCACCCACAUA  AGG UGUGUGGGUGGGUGUAU  3' U 5' | 5' G UC C 3'  CCAC C CCCACCCACAUA  GGUG G GGGUGGGUGUAU  3' UA U U 5' |

Supplementary Table 4 – Variants of CODEMIR-56 and 76 without 7 G motifs.

|  | Duplex | mRNA binding (RNA hybrid) |
| --- | --- | --- |
| CODEMIR-56 | Passenger  ACACCCCCCCACCCACAUAUU  UCUGUGGGGGGGUGGGUGUAU  Guide | VEGF 5' U A C 3'  AGACAC CCCACCCACAUA  UCUGUG GGGUGGGUGUAU  3' GGG 5' |
|  |  | ICAM-1 5' U C C 3'  AG CACCUCCCCACCCACAUA  UC GUGGGGGGGUGGGUGUAU  3' U 5' |
| CODEMIR-76 | Passenger  CGACCCCCCCACCCACAUAUU  UAGCUGGGGGGGUGGGUGUAU  Guide | VEGF 5' A ACA C 3'  GAC CCCACCCACAUA  CUG GGGUGGGUGUAU  3' UAG GGG 5' |
|  |  | ICAM-1 5' C C 3'  ACCUCCCCACCCACAUA  UGGGGGGGUGGGUGUAU  3' UAGC 5' |
| CODEMIR-120 | Passenger  ACACCUCCCCACCCACAUAUU  UCUGUGG**A**GGGGUGGGUGUAU  Guide | VEGF 5' U A C 3'  AGACAC CCCACCCACAUA  UCUGUG GGGUGGGUGUAU  3' G**A**G 5' |
|  |  | ICAM-1 5' U C C 3'  AG CACCUCCCCACCCACAUA  UC GUGG**A**GGGGUGGGUGUAU  3' U 5' |
| CODEMIR-121 | Passenger  CGACCUCCCCACCCACAUAUU  UAGCUGG**A**GGGGUGGGUGUAU  Guide | VEGF 5' A ACA C 3'  GAC CCCACCCACAUA  CUG GGGUGGGUGUAU  3' UAG G**A**G 5' |
|  |  | ICAM-1 5' C C 3'  ACCUCCCCACCCACAUA  UGG**A**GGGGUGGGUGUAU  3' UAGC 5' |

**N.B. Red indicates nucleotide substitution relative to original sequence.**

Supplementary Table 5 – Variants of CODEMIR-1 with central mismatches to VEGF.

|  | Duplex | VEGF binding (RNA hybrid) |
| --- | --- | --- |
| CODEMIR-1 | Passenger  AGACUCACCCACCCACAUAUU  AAUCUGAGUGGGUGGGUGUAU  Guide | 5' G A C 3'  UAGAC CACCCACCCACAUA  AUCUG GUGGGUGGGUGUAU  3' A A 5' |
| siVAIC | Passenger  AGACACACCCACCCACAUAUU  UUUCUGUGUGGGUGGGUGUAU  Guide | 5' GU C 3'  AGACACACCCACCCACAUA  UCUGUGUGGGUGGGUGUAU  3' UU 5' |
| CODEMIR-45 | Passenger  AGACACACCCUCCCACAUAUU  UUUCUGUGUGGGAGGGUGUAU  Guide | 5' U A C 3'  AGACACACCC CCCACAUA  UCUGUGUGGG GGGUGUAU  3' UU A 5' |
| CODEMIR-46 | Passenger  AGACACACCGACCCACAUAUU  UUUCUGUGUGGCUGGGUGUAU  Guide | 5' U C C 3'  AGACACACC ACCCACAUA  UCUGUGUGG UGGGUGUAU  3' UU C 5' |
| CODEMIR-47 | Passenger  AGACACACGCACCCACAUAUU  UUUCUGUGUGCGUGGGUGUAU  Guide | 5' U C C 3'  AGACACAC CACCCACAUA  UCUGUGUG GUGGGUGUAU  3' UU C 5' |
| CODEMIR-48 | Passenger  AGACACAGCCACCCACAUAUU  UUUCUGUGUCGGUGGGUGUAU  Guide | 5' U C C 3'  AGACACA CCACCCACAUA  UCUGUGU GGUGGGUGUAU  3' UU C 5' |
| CODEMIR-49 | Passenger  AGACACACCGUCCCACAUAUU  UUUCUGUGUGGCAGGGUGUAU  Guide | 5' U CA C 3'  AGACACACC CCCACAUA  UCUGUGUGG GGGUGUAU  3' UU CA 5' |
| CODEMIR-50 | Passenger  AGACACACGGACCCACAUAUU  UUUCUGUGUGCCUGGGUGUAU  Guide | 5' U CC C 3'  AGACACAC ACCCACAUA  UCUGUGUG UGGGUGUAU  3' UU CC 5' |
| CODEMIR-51 | Passenger  AGACACAGGCACCCACAUAUU  UUUCUGUGUCCGUGGGUGUAU  Guide | 5' U CC C 3'  AGACACA CACCCACAUA  UCUGUGU GUGGGUGUAU  3' UU CC 5' |

Supplementary Table 6 – Variants of CODEMIR-1 incorporating inosine bases.

|  | Duplex | mRNA binding (RNA hybrid) |
| --- | --- | --- |
| CODEMIR-1 | Passenger  AGACUCACCCACCCACAUAUU  AAUCUGAGUGGGUGGGUGUAU  Guide | VEGF 5' G A C 3'  UAGAC CACCCACCCACAUA  AUCUG GUGGGUGGGUGUAU  3' A A 5' |
|  |  | ICAM 5' G CCAC C 3'  UUAG CUC CCCACCCACAUA  AAUC GAG GGGUGGGUGUAU  3' U U 5' |
|  |  |  |
| CODEMIR-100 | Passenger  AGACUCACCCACCCACAUAUU  AAUCUGAGIGGGUGGGUGUAU  Guide | VEGF 5' G A C 3'  UAGAC CACCCACCCACAUA  AUCUG GIGGGUGGGUGUAU  3' A A 5' |
|  |  | ICAM 5' G CC C C 3'  UUAG AC UCCCCACCCACAUA  AAUC UG GIGGGUGGGUGUAU  3' A 5' |
| CODEMIR-101 | Passenger  AGACUCACCCACCCACAUAUU  AAUCUGIGUGGGUGGGUGUAU  Guide | VEGF 5' G C 3'  UAGACACACCCACCCACAUA  AUCUGIGUGGGUGGGUGUAU  3' A 5' |
|  |  | ICAM 5' G CC C C 3'  UUAG ACCU CCCACCCACAUA  AAUC UGIG GGGUGGGUGUAU  3' U 5' |
| CODEMIR-102 | Passenger  AGACUCACCCACCCACAUAUU  AAUCUGIGIGGGUGGGUGUAU  Guide | VEGF 5' G C 3'  UAGACACACCCACCCACAUA  AUCUGIGIGGGUGGGUGUAU  3' A 5' |
|  |  | ICAM 5' G CC C 3'  UUAG ACCUCCCCACCCACAUA  AAUC UGIGIGGGUGGGUGUAU  3' 5' |

I indicates inosine base

Supplementary Table 7 – Variants of CODEMIR-1 with extended seed.

|  | CODEMIR duplex | Predicted binding (RNA Hybrid) |
| --- | --- | --- |
| CODEMIR-1 | Passenger  AGACUCACCCACCCACAUAUU  AAUCUGAGUGGGUGGGUGUAU  Guide | VEGF 5' G A C 3'  UAGAC CACCCACCCACAUA  AUCUG GUGGGUGGGUGUAU  3' A A 5' |
|  |  | ICAM 5' G CCAC C 3'  UUAG CUC CCCACCCACAUA  AAUC GAG GGGUGGGUGUAU  3' U U 5' |
| CODEMIR-11 | Passenger  GACUCACCCACCCACAUACUU  AUCUGAGUGGGUGGGUGUAUG  Guide | VEGF 5' G A A 3'  UAGAC CACCCACCCACAUAC  AUCUG GUGGGUGGGUGUAUG  3' A 5' |
|  |  | ICAM 5' U CCAC A 3'  UAG CUC CCCACCCACAUAC  AUC GAG GGGUGGGUGUAUG  3' U U 5' |
| CODEMIR-12 | Passenger  ACUCACCCACCCACAUACAUU  UCUGAGUGGGUGGGUGUAUGU  Guide | VEGF 5' U A U 3'  AGAC CACCCACCCACAUACA  UCUG GUGGGUGGGUGUAUGU  3' A 5' |
|  |  | ICAM 5' C U 3'  CUC CCCACCCACAUACA  GAG GGGUGGGUGUAUGU  3' UCU U 5' |
| CODEMIR-13 | Passenger  CUCACCCACCCACAUACAUUU  CUGAGUGGGUGGGUGUAUGUA  Guide | VEGF 5' A A A 3'  GAC CACCCACCCACAUACAU  CUG GUGGGUGGGUGUAUGUA  3' A 5' |
|  |  | ICAM 5' C U 3'  CUC CCCACCCACAUACAU  GAG GGGUGGGUGUAUGUA  3' CU U 5' |

**Supplementary Table 8 –** **VEGF-A 5’UTR, ORF and 3’UTR targeting duplexes.**

|  | **Duplex** | **VEGF Binding (RNA Hybrid)** |
| --- | --- | --- |
| **5’ UTR targeting** |  |  |
| V0110 | Passenger  GCUCGGUGGAGGAAUUUGAUU  CGCGAGCCACCUCCUUAAACU  Guide | VEGF 5' G CU U 3'  GCGCUCGGUG GGAAUUUGA  CGCGAGCCAC CCUUAAACU  3' CU 5' |
| V0241 | Passenger  GCCGACGGGAUGGGGAGAUUU  CCCGGCUGCCCUACCCCUCUA  Guide | VEGF 5' C CU U 3'  GGGCCGACGG UGGGGAGAU  CCCGGCUGCC ACCCCUCUA  3' CU 5' |
| V0272 | Passenger  GGAAAUCAGAGUGGAUUUUUU  GGGGUUUAGUCUCACCUAAAA  Guide | VEGF 5' U CU G 3'  CCCCAAAUCA GUGGAUUUU  GGGGUUUAGU CACCUAAAA  3' CU 5' |
| **ORF targeting** |  |  |
| V0634 | Passenger  GCGUCGCAGAGAAACUUUUUU  GCCGCAGCGUCUCUUUGAAAA  Guide | VEGF 5' G CU C 3'  CGGCGUCGCA GAAACUUUU  GCCGCAGCGU CUUUGAAAA  3' CU 5' |
| V1173 | Passenger  CGCAGCUAGAGCCAUGGAAUU  UCGCGUCGAUCUCGGUAGGUU  Guide | VEGF 5' C CU U 3'  AGCGCAGCUA GCCAUCCAA  UCGCGUCGAU CGGUAGGUU  3' CU 5' |
| V1236 | Passenger  GAGUACAUGAUCAAGCCAUUU  AGCUCAUGUACUAGUUCGGUA  Guide | VEGF 5' A CU C 3'  UCGAGUACAU UCAAGCCAU  AGCUCAUGUA AGUUCGGUA  3' CU 5' |
| V1387 | Passenger  UGAGCUUCGAACAGCACAAUU  CUACUCGAAGCUUGUCGUGUU  Guide | VEGF 5' A CU C 3'  GAUGAGCUUC ACAGCACAA  CUACUCGAAG UGUCGUGUU  3' CU 5' |
| V1549 | Passenger  CGAGGCAGGAUGAGUUAAAUU  CCGCUCCGUCCUACUCAAUUU  Guide | VEGF 5' A CU C 3'  GGCGAGGCAG UGAGUUAAA  CCGCUCCGUC ACUCAAUUU  3' CU 5' |
| **3’ UTR targeting** |  |  |
| V1645 | Passenger  CCAGAUCUGACACCAGGAAUU  UUGGUCUAGACUGUGGUCCUU  Guide | VEGF 5' G CU A 3'  AACCAGAUCU CACCAGGAA  UUGGUCUAGA GUGGUCCUU  3' CU 5' |
| V1797 | Passenger  GGGCGAGAGACCGGCGGAAUU  CUCCCGCUCUCUGGCCGCCUU  Guide | VEGF 5' G CU G 3'  GAGGGCGAGA CCGGCGGAA  CUCCCGCUCU GGCCGCCUU  3' CU 5' |
| V1938 | Passenger  AGACACACGGACCCACAUAUU  UUUCUGUGUGCCUGGGUGUAU  Guide | VEGF 5' U CC C 3'  AGACACAC ACCCACAUA  UCUGUGUG UGGGUGUAU  3' UU CC 5' |
| V2203 | Passenger  CCUCUCCCGAGCCCAGGAAUU  CAGGAGAGGGCUCGGGUCCUU  Guide | VEGF 5' G CU U 3'  GUCCUCUCCC GCCCAGGAA  CAGGAGAGGG CGGGUCCUU  3' CU 5' |
| V2826 | Passenger  AUACGGUAGAUAUUUAAUAUU  UAUAUGCCAUCUAUAAAUUAU  Guide | VEGF 5' U CU U 3'  AUAUACGGUA UAUUUAAUA  UAUAUGCCAU AUAAAUUAU  3' CU 5' |
| V2956 | Passenger  CUCUCUUGGACUCUUAUUUUU  GAGAGAGAACCUGAGAAUAAA  Guide | VEGF 5' G CU G 3'  CUCUCUCUUG CUCUUAUUU  GAGAGAGAAC GAGAAUAAA  3' CU 5' |
| V3034 | Passenger  GUCACUAGGAUAUCUUGAAUU  GUCAGUGAUCCUAUAGAACUU  Guide | VEGF 5' A CU C 3'  CAGUCACUAG UAUCUUGAA  GUCAGUGAUC AUAGAACUU  3' CU 5' |
| V3112 | Passenger  ACACAUUCGAUUGAAAUAAUU  CGUGUGUAAGCUAACUUUAUU  Guide | VEGF 5' A CU G 3'  GCACACAUUC UUGAAAUAA  CGUGUGUAAG AACUUUAUU  3' CU 5' |
