# Supplementary material for: Interfering ribonucleic acids that suppress expression of multiple unrelated genes
Source: BMC Biotechnol. 2009 Jun 16;9:57. doi: 10.1186/1472-6750-9-57 (PMC2706242; doi:10.1186/1472-6750-9-57)
Supplement: Additional file 2 — Additional figures. Figures and legends demonstrating supporting data for the study. [file 1472-6750-9-57-S2.doc]

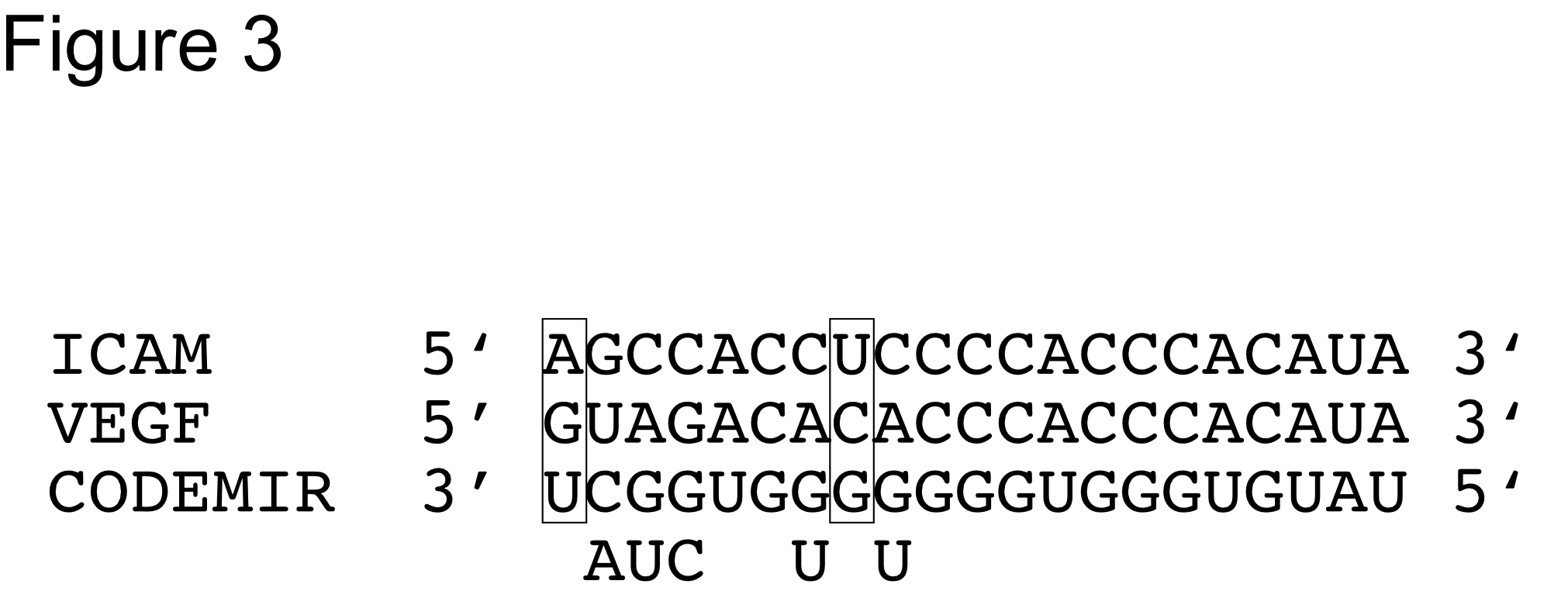


**Figure S1.**  Schematic illustration of the design of the 32 variants of CODEMIR-1 aligned to the *VEGF-A* and *ICAM-1* mRNAs. Mismatch sites are indicated by the presence of the alternative base below the indicated guide strand sequence, wobble base positions are indicated by boxes.


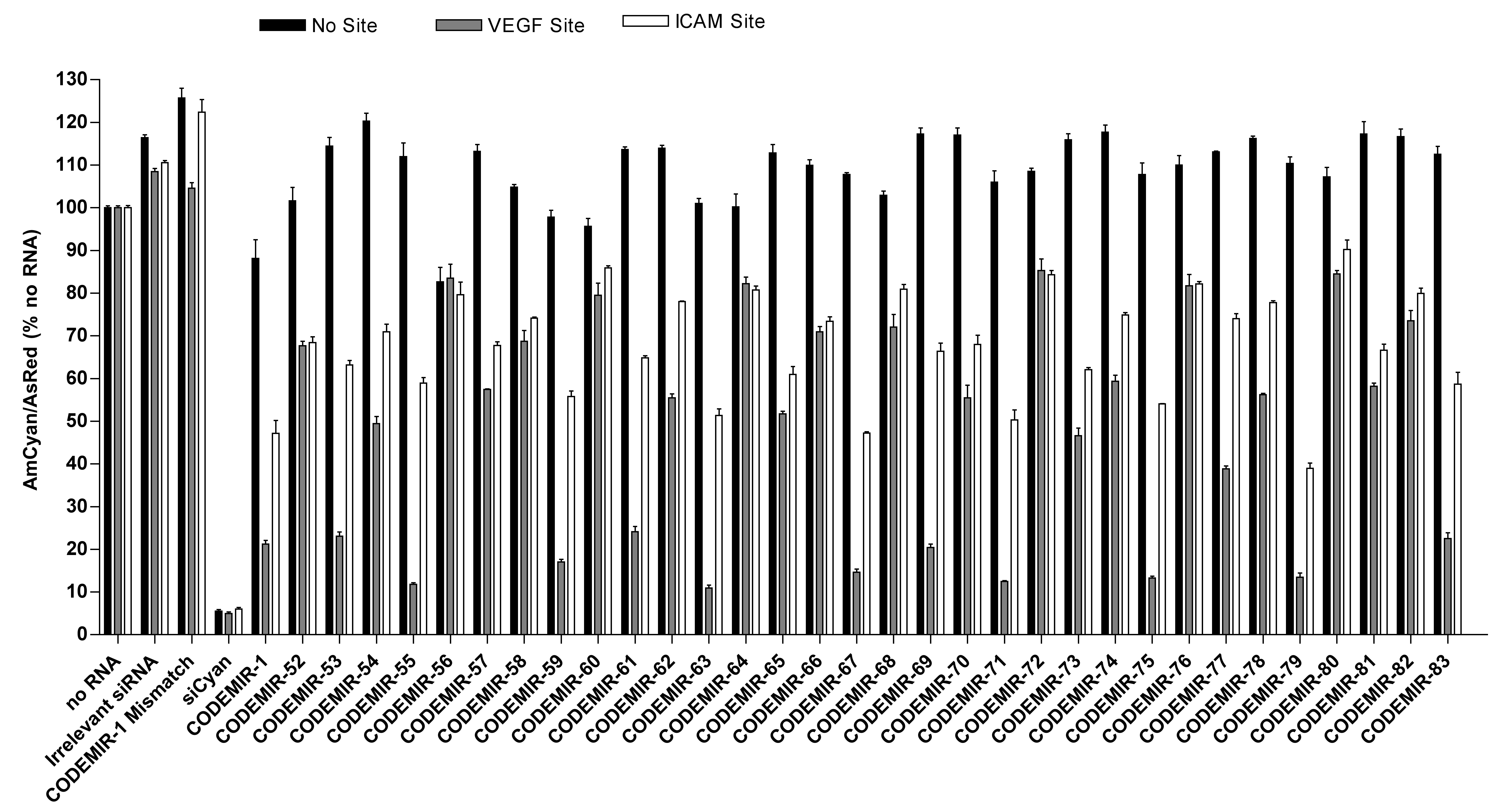


**Figure S2.** Normalised fluorescence of ARPE-19 cells transfected with AmCyan/CODEMIR-1 reporter and AsRed plasmids and CODEMIR-1 tail variants. ARPE-19 cells were co-transfected with 1 μg of the plasmids and 40 nM indicated RNA duplexes. Fluorescence was assessed by FACS 48 hours post-transfection. Transfections were performed in triplicate. Error bars indicate standard deviation


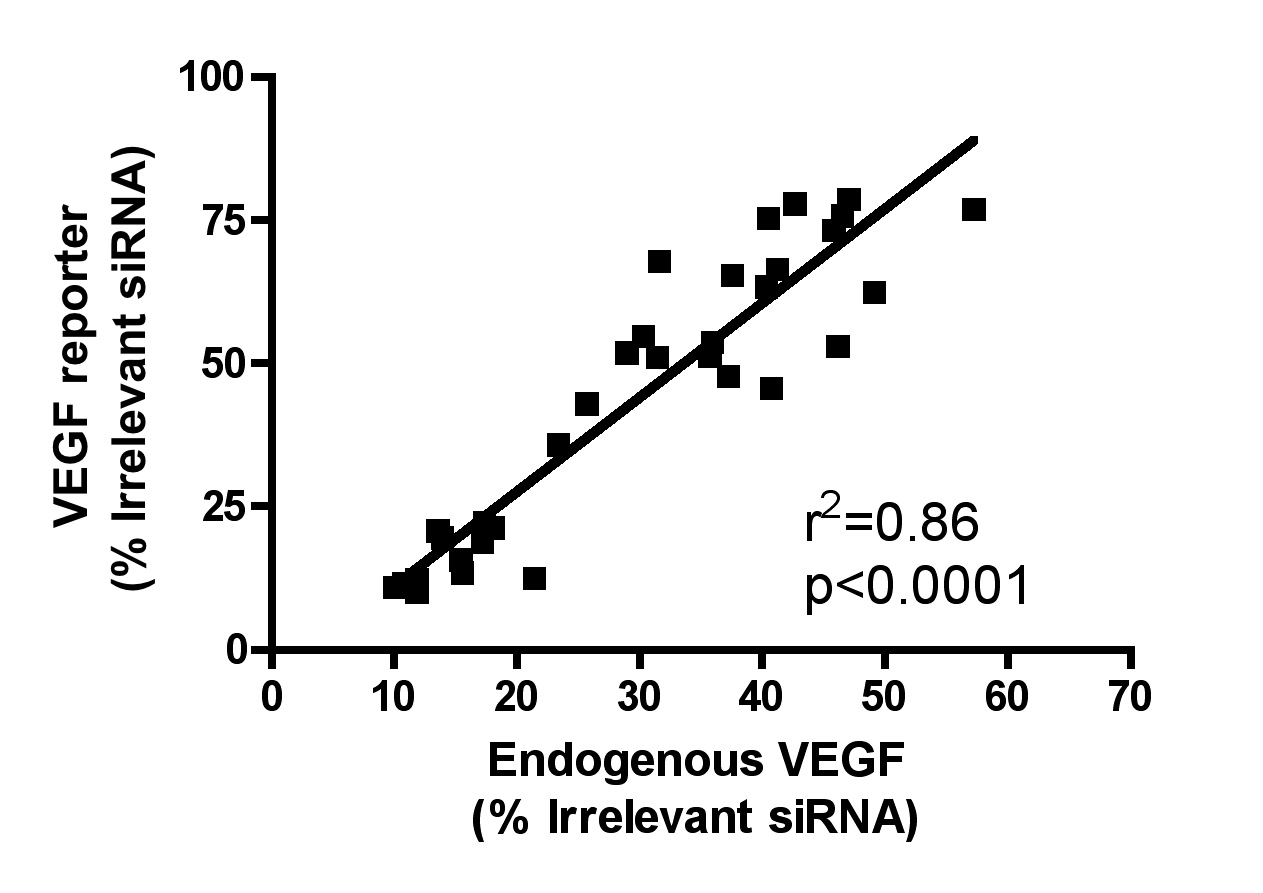


**Figure S3.** Correlation between endogenous *VEGF-A* and reporter suppression by CODEMIR-1 and CODEMIR-1 tail variants.


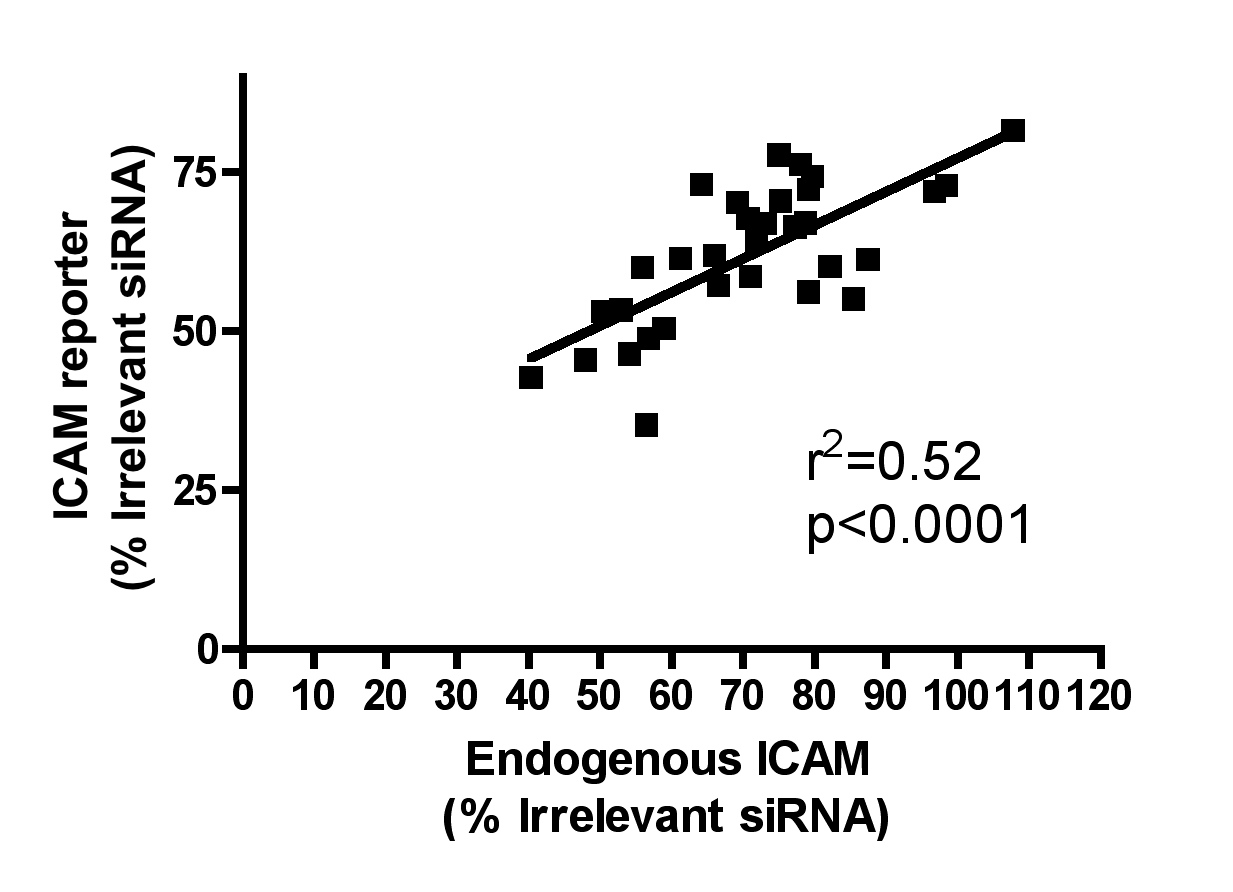


**Figure S4.** Correlation between endogenous *ICAM-1* and reporter suppression by CODEMIR-1 and CODEMIR-1 tail variants.


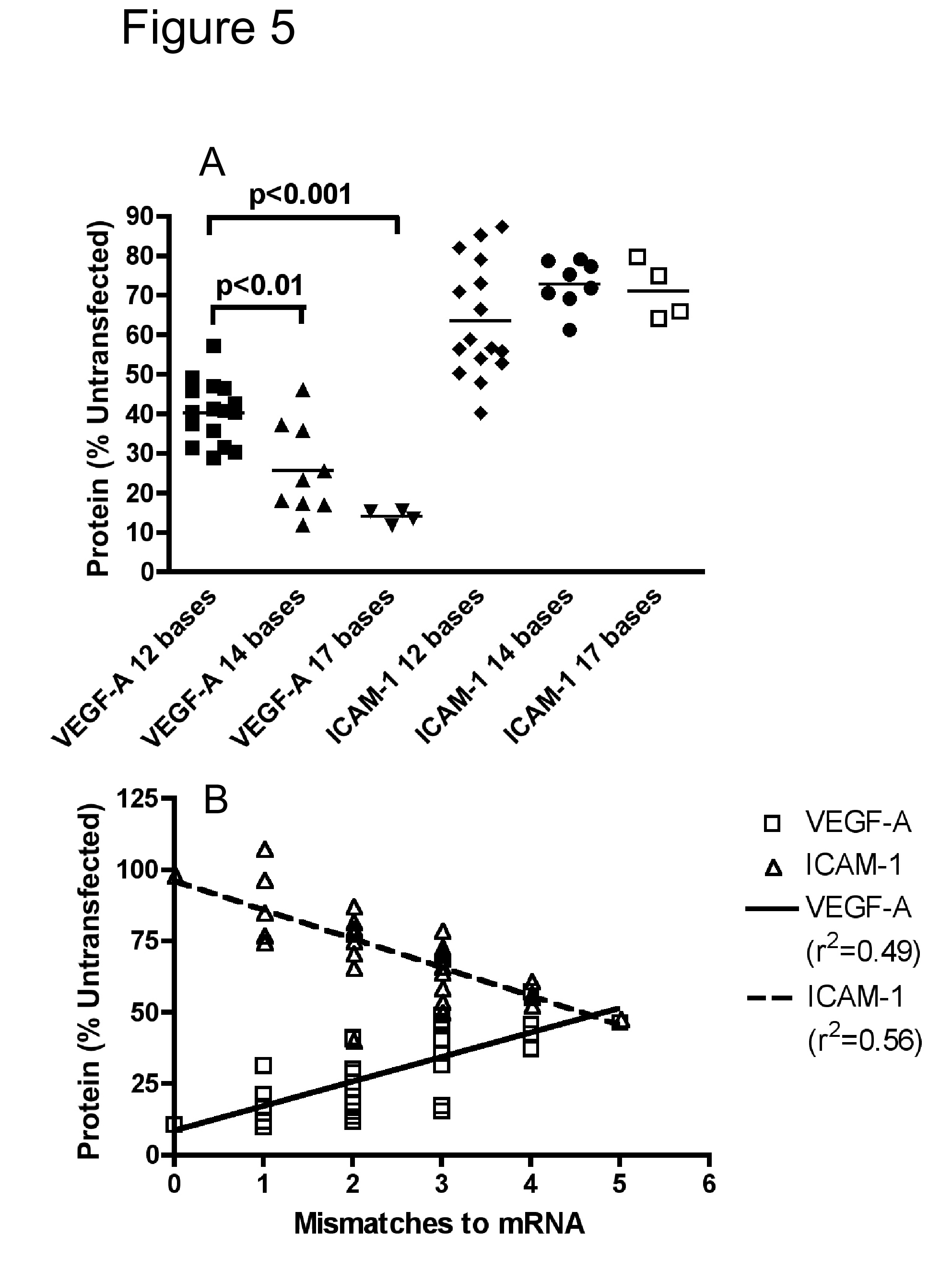


**Figure S5.** (A) Relationship between length of contiguous perfect complementarity (between guide strand and mRNA target as measured from the 5’ end of the guide strand) and target suppression for the 32 variants assayed in Figure 3. (B) Relationship between total number of mismatches (between guide strand and target mRNA) and target suppression for the 32 variants assayed in Figure 4. The slopes of both regression lines were significantly non-zero (p<0.0001).


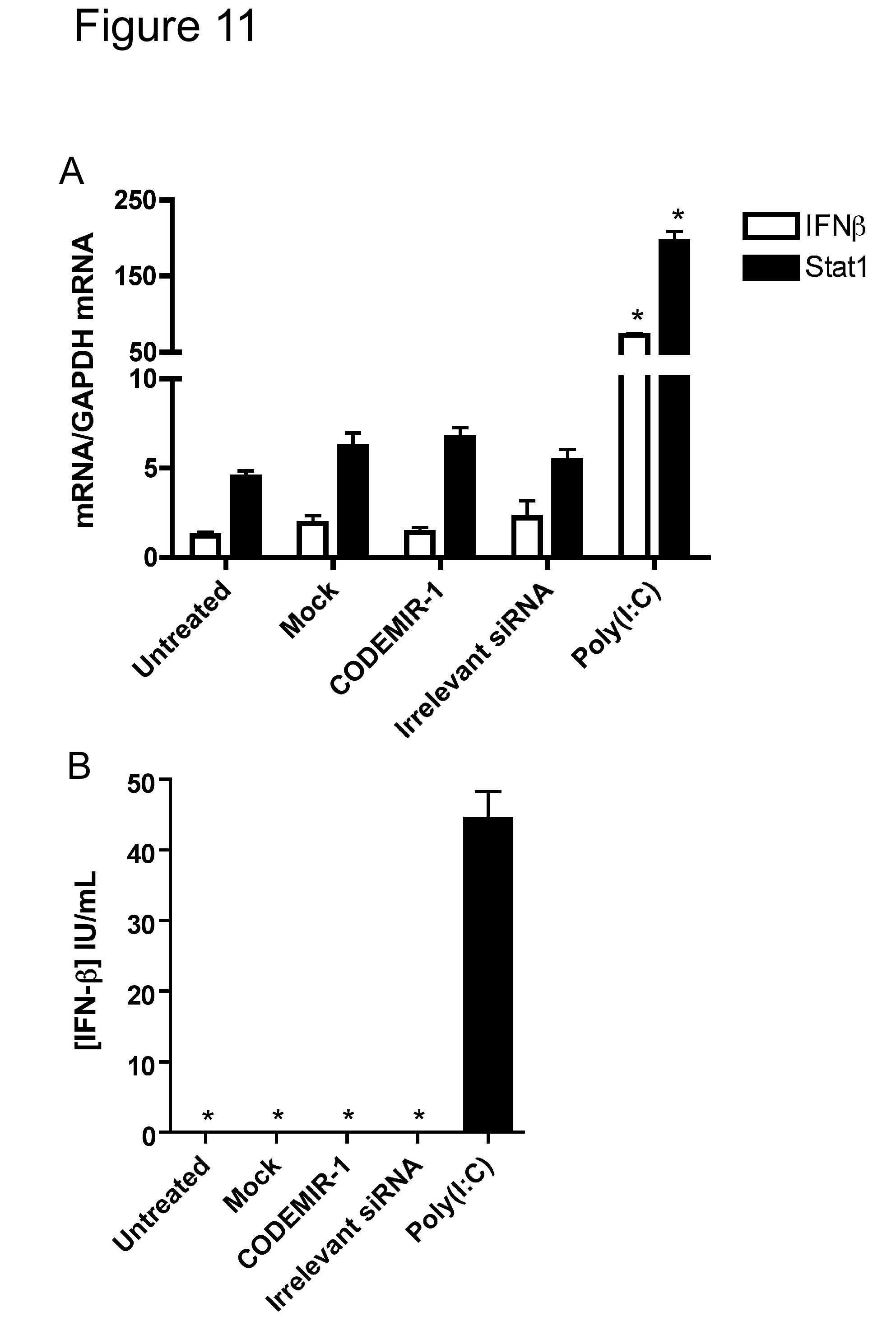


**Figure S6**. (A) Expression of *IFNβ* and *STAT1* mRNA normalised to *GAPDH*. ARPE19 cells were transfected with 100 nM dsRNA or 50 ng/mL poly(I:C) in Lipofectamine 2000. The QuantiGene® assay was performed 24 hours post-transfection. Each bar represents the mean of triplicate samples. Error bars indicate standard deviation (*p<0.001 as compared to untreated cells - determined by two-way ANOVA using a Bonferroni post-test). (B) IFNβ secretion by ARPE19 cells transfected with various RNA. Cells were transfected with 120 nM indicated RNAs or 50 ng/mL poly(I:C). IFNβ in cell supernatants was assayed by ELISA 48 hours post-transfection. Each bar represents the mean of triplicate samples. Error bars indicate standard deviation. * indicates undetectable (limit of detection = 1 IU/mL).
